# Synthetic host defense peptide inhibits SARS-CoV-2 replication *in vitro*

**DOI:** 10.1128/aac.01700-24

**Published:** 2025-06-23

**Authors:** Rhodri Harfoot, Blair Lawley, Leonor C. Hernández, Joanna Kuang, Francesca R. Hills, Shubhra Sinha, Margot J. M. Allais, Tom W. Bird, Cody P. Hird, John A. Taylor, Mihnea Bostina, Davide Comoletti, Evan F. Haney, Robert E. W. Hancock, Daniel Pletzer, Miguel E. Quiñones-Mateu

**Affiliations:** 1 Department of Microbiology and Immunology, School of Biomedical Sciences, University of Otago 626306, Dunedin, New Zealand; 2 Department of Pediatric Dentistry, Schulich School of Medicine and Dentistry, Western University 70384, London, Ontario, Canada; 3 School of Biomedical Sciences, Victoria University of Wellington 8491, Wellington, New Zealand; 4 Institute of Environmental Science and Research, Wellington, New Zealand; 5 Department of Pathology and Laboratory Medicine, Schulich School of Medicine and Dentistry 210508, London, Ontario, Canada; 6 School of Biological Sciences, The University of Auckland 99026, Auckland, New Zealand; 7 Centre for Microbial Diseases and Immunity Research, Department of Microbiology and Immunology, University of British Columbia 270355, Vancouver, British Columbia, Canada; IrsiCaixa Institut de Recerca de la Sida, Barcelona, Spain

**Keywords:** SARS-CoV-2, COVID-19, host defense peptides, antiviral, influenza virus

## Abstract

Although myriads of potential antiviral agents have been tested against SARS-CoV-2, only a handful have proven to be effective in clinical trials. During the COVID-19 pandemic, many known or novel peptides were evaluated for their ability to inhibit SARS-CoV-2 replication; however, testing of D-enantiomers that resist body and viral proteases has been limited. Here, we characterized the ability of D-3006, a D-enantiomeric synthetic host defense peptide, to inhibit SARS-CoV-2 replication *in vitro*. A battery of authentic SARS-CoV-2 variants (ancestral, Mu, Delta, and Omicron BA.1) and a comprehensive panel of β-coronavirus spike pseudotyped lentiviruses were used to demonstrate that D-3006 safely (CC_50_value = 430 µg/mL) blocked spike-mediated entry (EC_50_ values ranging from 1.57 to 5.37 µg/mL) and also had synergistic anti-SARS-CoV-2 activity *in vitro* when combined with the viral polymerase inhibitor remdesivir. We also showed that D-3006 inhibited influenza A virus (H1N1) replication *in vitro*, suggesting that this synthetic host defense peptide could have potential broad antiviral activity against multiple enveloped viruses. These data, together with negative-stain transmission electron microscopy analysis, suggest that the mechanism of action of D-3006 is associated with non-specific binding to the viral membrane, most likely causing virus aggregation and interfering with virus attachment and entry. The potential broad-spectrum antiviral activity of D-3006, its innate resistance to host proteases, as well as the possibility of being used in combination with other antiviral drugs suggest that this host synthetic peptide could be developed as a candidate for the treatment of SARS-CoV-2 and/or other respiratory viral infections.

## INTRODUCTION

SARS-CoV-2, the etiologic agent of the Coronavirus Disease of 2019 (COVID-19) (1), recently tested the world’s capacity to respond to a viral pandemic. Although early in the pandemic, public health and non-pharmaceutical interventions played a role in decreasing the number of new SARS-CoV-2 infections in certain countries (2–4), COVID-19 vaccines were instrumental in the fight against this new coronavirus (5). As of 25 March 2022, close to 350 COVID-19 vaccine candidates were either in pre-clinical or clinical development (https://www.who.int/publications/m/item/draft-landscape-of-covid-19-candidate-vaccines, accessed on 28 March 2022) based on established or novel methodologies, that is, from classical inactivated virus, protein subunits, and viral vectors to revolutionary mRNA technologies (3, 6). This colossal effort led to the development of safe and effective COVID-19 vaccines in record time, with nine COVID-19 vaccines included in the World Health Organization (WHO) Emergency Use Listing (https://covid19.who.int/, accessed on 28 March 2022) and over 13 billion vaccine doses administered worldwide (7).

Although the roll-out and widespread use of vaccines have significantly reduced the number of hospitalizations and, more importantly, deaths associated with COVID-19 (5), there still is a clear need for therapeutic approaches to prevent and/or manage acute SARS-CoV-2 infections. Similar to vaccine development, a massive effort to identify potent anti-SARS-CoV-2 agents started in early 2020 (8). Pharmaceutical and biotech companies, together with academic research institutions, have screened and tested a massive number of compounds to block SARS-CoV-2 replication. Starting with repurposed drugs approved by the U.S. Food and Drug Administration (FDA), the list of potential therapeutic options for the novel coronavirus has included anything from monoclonal antibodies (mAbs), recombinant human angiotensin-converting enzyme 2 (hACE2), natural products, or small molecules (typically organic compounds with low molecular weight) targeting different steps in the virus life cycle (e.g., entry, the RNA-dependent RNA polymerase [RdRp], or the 3CLpro and PLpro proteases) or even host targets (e.g., cellular enzyme transmembrane protease serine 2 [TMPRSS2], interferon therapies) (8–10). Despite the large number of pre-clinical and clinical studies, to date, only the nucleoside analogs VEKLURY (remdesivir, GS-5734) and LAGEVRIO (molnupiravir, EIDD-1931), the protease inhibitor PAXLOVID (nirmatrelvir, PF-07321332 plus ritonavir), and a handful of neutralizing mAbs have been approved by the FDA for the treatment of COVID-19 (8, 10).

Host defense peptides (HDPs), integral members of the innate host immune response, have been shown to protect against microbial invasion at the mucosal barrier (11–15), with some being able to also block virus replication (11, 16–20). These natural cationic (net positive charge) peptides derived from multiple living organisms, as well as rationally designed molecules related to the native peptides, have been tested as potential inhibitors of multiple DNA and/or RNA viruses, for example, cytomegalovirus (CMV), herpes simplex virus types 1 and 2 (HSV-1 and 2), human papillomavirus (HPV), human immunodeficiency virus (HIV), influenza A virus (IAV), respiratory syncytial virus (RSV), SARS-CoV, and MERS-CoV (19–27). Not surprisingly, a number of these peptides have also been evaluated for their ability to inhibit SARS-CoV-2 replication (28). For example, human cathelicidin LL-37 (29, 30), human neutrophil peptide 1 (HNP1) (31), human α-defensin 5 (HD5) (30–32), human β-defensin 2 (hβD-2) (33), fungal (34) or frog-derived (35) defensins, as well as the retrocyclin RC-101, a peptide synthesized based on human θ defensin pseudogenes (31, 36), have been shown to block viral entry using pseudotyped viruses expressing SARS-CoV-2 spike proteins and/or replication-competent (authentic) SARS-CoV-2. In addition, *de novo*-designed synthetic peptides modeled after HDPs, such as brilacidin (37), the antibacterial peptide DP7 (38), and the defensin-like peptide P9R, derived from mouse β-defensin 4 (24), inhibited SARS-CoV-2 replication *in vitro*. Some of these HDPs have also been shown to block SARS-CoV-2 transmission in animal models by interfering with the interaction of the SARS-CoV-2 receptor binding domain (RBD) with the hACE2 receptor or acting as fusion inhibitors (24, 28, 38–40). However, most of these peptides demonstrated modest activity, and, more importantly, most of them are susceptible to body (and possibly viral) proteases, which could strongly impact their antiviral activity.

The most common antimicrobial mechanism of action of host defense peptides seems to be associated with the disruption of lipid bilayer membranes, which can lead to cell death, although some HDPs can also target proteins in cell membranes and/or interfere with a number of intracellular functions, including ribosome flocculation or blocking the stringent stress response mediator molecule guanosine tetraphosphate (ppGpp) (15, 41–43). Our group has a long history of designing and characterizing synthetic HDPs to target bacterial pathogens and their biofilm growth mode (41, 44, 45). A lead candidate is the L-enantiomeric innate defense regulator 1018 (IDR-1018), a synthetic 12-amino acid peptide distantly related to bactenecin, a HDP found in bovine neutrophils (41, 46) that has also been shown to modulate the immune system (44, 47). Interestingly, IDR-1018 has also shown antiviral activity, that is, blocking the replication of HSV-2 in Vero cells and reducing HSV-2 infectivity in mice (27). As mentioned above, one limitation of natural or synthetic HDPs, such as IDR-1018, is that they are based on L-amino acids, which make them vulnerable to proteolytic enzymes (48). Conversely, D-enantiomeric peptides are less susceptible to host proteases, which extends their half-life while maintaining their biological activity (41). For example, DJK-5 is a potent D-enantiomeric peptide with broad-spectrum antibiofilm activity (49), including oral and *Pseudomonas aeruginosa* biofilms (50, 51), with therapeutic potential against biofilm-associated skin infections (45, 52, 53).

In this study, we assessed the ability of four synthetic host defense peptides to inhibit SARS-CoV-2 replication *in vitro*, including the bactenecin-related IDR-1018 and three D-enantiomeric peptides: DJK-5 and two other D-enantiomers of anti-biofilm peptides (D-3006 and D-3007). We evaluated their cellular and anti-SARS-CoV-2 profile in different susceptible cell lines, showing that the HDPs inhibited SARS-CoV-2 replication, with D-3006 being the most potent candidate. Using a comprehensive panel of β-coronavirus spike pseudotyped viruses and authentic SARS-CoV-2 strains, we showed that D-3006 blocked spike-mediated entry and has synergistic anti-SARS-CoV-2 activity *in vitro* when combined with remdesivir. Interestingly, preliminary results with influenza A virus (H1N1) seem to suggest that this D-enantiomeric peptide could have potential broad antiviral activity against multiple enveloped viruses.

## MATERIALS AND METHODS

### Cells and viruses

Vero (CCL-81 ATCC, Manassas, VA, USA), Caco-2 (HTB-37 ATCC), Calu-3 (HTB-55 ATCC), Huh-7 (CellBank Australia, Westmead, NSW, Australia), and MDCK (CCL-34 ATCC) cells were grown in high glucose DMEM (Thermo Fisher Scientific, Waltham, MA, USA) supplemented with 5% fetal bovine serum (FBS, Cellgro Mediatech, Manassas, VA, USA), 100 units/mL of penicillin, and 100 µg/mL of streptomycin (Thermo Fisher Scientific). VeroE6/TMPRSS2 (54) cells were purchased from the Japanese Collection of Research Bioresources Cell Bank (Osaka, Japan) and maintained as described above for Vero cells with the addition of 1 µg/mL of Geneticin (Thermo Fisher Scientific). HEK293 cells lacking N-acetylglucosaminyltransferase I (GnTI-) activity (HEK293 GnTI- CRL-3022 ATCC) were cultured in DMEM supplemented with 5% FBS and glutamine at 37°C and 5% CO_2_. HEK293T cells expressing human angiotensin-converting enzyme 2 (HEK293T-hACE2 cells, BEI Resources, Manassas, VA, USA) were cultured in DMEM supplemented with 10% FBS, 100 units/mL of penicillin, 100 µg/mL of streptomycin, and 2 µg/mL of puromycin (Thermo Fisher Scientific). HEK293-hDPP4 cells expressing the human dipeptidyl peptidase-4 (DPP4) gene (M.J.M. Allais, B. Lawley, and M.E. Quiñones-Mateu, unpublished data) were generated by stable transfection of HEK293 cells with plasmid pLEX307-DPP4-puro (a gift from Alejandro Chavez & Sho Iketani, Addgene plasmid #158451, Addgene, Watertown, MA, USA) and cultured as described above for HEK293T-hACE2 cells. ELVIRA Flu A cells (55) (Quidel Corporation, San Diego, CA, USA) were grown as described above for Vero cells but in fibronectin-coated plates (1 µg/cm^2^), with the addition of 150 µg/mL of hygromycin B (Gibco Thermo Fisher Scientific). The SARS-CoV-2 hCoV-19/New Zealand/NZ1_patient/2020 strain was originally isolated from a COVID-19 patient by our group (56). The SARS-CoV-2-Nluc (expressing NanoLuc, an ATP-independent luciferase) and SARS-CoV-2-mNG (mNeonGreen, a monomeric yellow-green fluorescent protein) were kindly provided by Dr. Pei-Yong Shi (University of Texas Medical Branch, Galveston, TX, USA). The influenza A virus (IAV) A/Mallard/Alberta/287/2012 (H1N1) strain was donated by Dr. Richard J. Webby (St. Jude Children’s Research Hospital, Memphis, TN, USA). Virus stocks were prepared by growing SARS-CoV-2 and IAV in VeroE6/TMPRSS2 and MDCK cells, respectively. Cell-culture supernatant was harvested, clarified by centrifugation at 1,500 rpm for 10 min, filtered through a 0.45 µm steriflip filter (Merck Millipore, Burlington, MA, USA), aliquoted, and stored at −80°C until further use. Tissue culture dose for 50% infectivity (TCID_50_) was determined in triplicate for each serially diluted virus using the Reed and Muench method (57), and viral titers were expressed as infectious units per milliliter (IU/mL).

### Synthetic host defense peptides

A 12-amino acid peptide distantly related to bovine bactenecin (IDR-1018, VRLIVAVRIWRR-NH_2_ (47)) manufactured by CPC Scientific and three conceptually related D-enantiomeric peptides (DJK-5, vqwrairvrvir-NH_2_ (49), D-3006, iwlrlkvvlkrk-NH_2_, and D-3007, vlkikvkiwvvk-NH_2_, this study) were synthesized using solid-phase 9-fluorenylmethoxy carbonyl (Fmoc) chemistry and purified to ~95% using reverse-phase high performance liquid chromatography. Peptide identity was confirmed by mass spectrometry and stock solutions, prepared in sterile water, and stored at −20°C until further use.

### Soluble human ACE2 and RBD

The cDNA insert of the human angiotensin-converting enzyme 2 (ACE2) extracellular domain (amino acids 18–740, Uniprot ID Q9BYF1) was synthesized and sequence verified by Gene Universal (Newark, DE, USA). The amino acid sequence between residues 329 and 537 of the SARS-CoV-2 Wuhan spike protein was used to create a soluble RBD fragment. The coding sequence of each construct (hACE2 and SARS-CoV-2 RBD) was cloned by 5’ *Not*I and 3’ *Xba*I into a modified pCMV6-XL4 vector in frame with an N-terminal DYKDDDDK peptide and a C-terminal human Fc fragment (58), generating plasmid hACE2-Fc (archived as Fc602) and the RBD-Fc (archived as Fc605). hACE2-AP was generated in the same way except that it was in frame with an alkaline phosphatase enzyme A. Stable cell lines expressing either plasmid (hACE2 or RBD) were made by co-transfecting HEK293 GnTI- cells with the hACE2-Fc or RBD-Fc and empty pcDNA3.1 plasmids (at a 3:1 ratio) and selected for Geneticin-resistant cells (G418, Thermo Fisher Scientific) by growing the cells in DMEM supplemented with 5% FBS and 500 µg/mL of G418. Geneticin-resistant cell clones were isolated using Pyrex cloning rings (Merck Sigma-Aldrich, St Louis, MO, USA), and stable expression of the hACE2 or RBD-Fc proteins was verified using western blot (59). Higher-expressing cell clones with high expression of the hACE2 protein were amplified and used in large-scale protein production as described (60)(61). Briefly, HEK293 GnTI- cells stably expressing hACE2 or RBD-Fc were grown in triple-layer cell culture flasks with regular collection and replenishment of the cell culture medium containing 2%–5% FBS. Secreted proteins were purified via affinity chromatography, using anti-FLAG M2 affinity resin (Merck Sigma-Aldrich) to capture the N-terminal DYKDDDDK peptide. The saturated resin was washed (50 mM Tris pH 7.4, 450 mM NaCl), equilibrated into 1× phosphate-buffered saline (PBS), and the protein was eluted in PBS added with 100 µg/mL of DYKDDDDK peptide (Thermo Fisher Scientific). The eluted protein was then concentrated to 3–6 mg/mL using concentrators with the appropriate molecular weight cutoff prior to being flash-frozen in liquid nitrogen and stored at −80°C.

### Cellular toxicity assay

The potential cytotoxic effect of the synthetic peptides (D-3006, D-3007, DJK-5, or IDR-1018) and control agents (melittin or dimethyl sulfoxide [DMSO], Merck Sigma-Aldrich) was determined by quantifying both cell viability and cellular proliferation in different cell lines using the XTT [2,3-Bis-(2-Methoxy-4-Nitro-5-Sulfophenyl)−2H-Tetrazolium-5-Carboxanilide] colorimetric method in the Cell Proliferation Kit II (Merck Sigma-Aldrich). Briefly, 2 × 10^4^ cells/well were seeded in a 96-well plate and incubated at 37°C, 5% CO_2_ overnight. The cell culture medium was replaced with fresh media containing empirically determined serial dilutions of the corresponding synthetic peptides or control agents in triplicate and incubated at 37°C, 5% CO_2_ for 48 h. The cell culture medium was then carefully removed, and the cells were washed twice with 1× PBS. Fifty microliters of fresh XTT labeling reagent (Cell Proliferation Kit II, Merck Sigma-Aldrich) were added to each well and incubated at 37°C, 5% CO_2_ for 4 h, and the absorbance was quantified at 570 nm in a Varioskan LUX multimode microplate reader (Thermo Fisher Scientific). The 50% cytotoxic concentration (CC_50_), defined as the concentration of a compound that caused cell death or inhibited cell proliferation by 50%, was calculated using GraphPad Prism v.9.2.0 (GraphPad Software, La Jolla, CA, USA).

### Drug susceptibility assay using replication-competent SARS-CoV-2

The susceptibility of authentic SARS-CoV-2 isolates (56) to the synthetic peptides (D-3006, D-3007, DJK-5, or IDR-1018) and remdesivir (RDV, GS-5734, Sapphire Bioscience, Waterloo, Australia) was evaluated in Vero or VeroE6/TMPRSS2 cells. Serial dilutions spanning empirically determined ranges of each synthetic peptide or RDV were added in triplicate to 96-well plates containing Vero or VeroE6/TMPRSS2 cells (20,000 cells/well) and incubated at 37°C, 5% CO_2_ for 2 h. Cells were then infected with SARS-CoV-2 at a MOI of 0.005 IU/cell for 1 h at 37°C, 5% CO_2_. After the virus inoculum was removed, the cells were washed twice, and the complete medium with the corresponding agent dilution was replenished. SARS-CoV-2 replication was quantified 72 h post-infection by CPE, RT-qPCR assay (62), or a cell protection assay based on the Pierce BCA Protein Assay Kit (Thermo Fisher Scientific) as described (56). Concentrations of the synthetic peptides or RDV required to inhibit SARS-CoV-2 replication by 50% (EC_50_) were calculated by plotting the percent inhibition of virus replication versus log_10_ drug concentration and fitting the inhibition curves to the data using nonlinear regression analysis (GraphPad Prism v.9.3.1, GraphPad Software).

### Time-of-drug addition assay

Confluent monolayers of VeroE6/TMPRSS2 cells in a 96-well plate were washed with 1× PBS and infected with the SARS-CoV-2 hCoV-19/New Zealand/NZ1_patient/2020 isolate at a MOI of 0.005 IU/cell. The synthetic peptides (D-3006, D-3007, DJK-5, or IDR-1018) or the control agents (shACE2, RDV, or a SARS-CoV-2 spike neutralizing antibody, rabbit mAb, cat. 40592-R0004, SinoBiological, Beijing, China) were added in triplicate at different time points pre-infection (−2 h), during (0 h), or post-infection (+2 h or +16 h) at a concentration equal to 10-fold of their respective EC_50_ values. Cells were washed twice with 1× PBS 1 h post-infection to remove unbound virus, replenishing the complete media in the absence of synthetic peptides or control agents. SARS-CoV-2 replication was quantified 72 h post-infection using a cell protection assay based on the Pierce BCA Protein Assay Kit (Thermo Fisher Scientific) as described (56).

### Generation of betacoronavirus spike-pseudotyped lentiviruses

Pseudotyped lentiviral particles incorporating the spike glycoprotein from SARS-CoV-2, SARS-CoV, or MERS-CoV with 19 amino acids removed from the carboxy-terminal were generated as described (63). Briefly, 4 × 10^5^ cells HEK293T cells/well were seeded in 6-well plates (3 mL of DMEM, 10% fetal bovine serum, 100 units/mL of penicillin, and 100 µg/mL of streptomycin, Thermo Fisher Scientific) and incubated at 37°C, 5% CO_2_ for 24 h. Cells were then co-transfected, using FuGENE HD Transfection Reagent (Promega, Madison, Wisconsin), with the following plasmids: (i) 1 mg of pHAGE-CMV-Luc2-IRES-ZsGreen-W, (ii) 0.22 mg of HDM-Hgpm2, (iii) 0.22 mg of pRC-CMV-Rev1b, (iv) 0.22 mg of HDM-tat1b, and (v) 0.37 mg of a plasmid containing a codon-optimized SARS-CoV-2, SARS-CoV, or MERS-CoV spike protein. In addition to the plasmid expressing the spike protein from the ancestral SARS-CoV-2 Wuhan isolate (1), that is, pcDNA3.1_spike_Δ19 (a gift from R). De Francesco, plasmid# 155297, Addgene, Watertown, MA), a series of codon-optimized spike glycoproteins from different betacoronaviruses were synthesized and cloned using 5’ *Not*I and 3’ *Xba*I into a modified pcDNA3.1 vector in frame with an N-terminal DYKDDDDK peptide to generate a comprehensive panel of pseudotyped viral particles containing the spike protein from SARS-CoV-2 B.1.1.7 (Alpha), B.1.351 (Beta), P.1 (Gamma), B.1.617.2 (Delta), B.1.429 (Epsilon), B.1.617.1 (Kappa), C.37 (Lambda), B.1.621 (Mu), B.1.525 (Eta), B.1.526 (Theta), and B.1.1.529 (Omicron BA.1) variants, as well as SARS-CoV and MERS-CoV. Plasmid pMD2.G, encoding the G envelope protein from Vesicular stomatitis virus (VSV-g, a gift from Didier Trono, plasmid #12259, Addgene, Watertown, MA), was used as a positive control. Cell culture media was changed 24 h after transfection, and the cell-free supernatant was collected at 48 h post-transfection, filtered through a 0.45-µm cellulose acetate filter (Ahlstrom Munksjo, Helsinki, Finland), and 1 mL aliquots were stored at −80°C until further use. The titer of the betacoronavirus spike pseudotyped lentiviruses was determined by infecting HEK293T-hACE2 cells (for all SARS-CoV-2 variants and SARS-CoV) or HEK293-hDPP4 cells (for MERS-CoV), and TCID_50_ values were determined as described above.

### Betacoronavirus spike-pseudotyped virus neutralization assay

The ability of the synthetic peptides (D-3006, D-3007, DJK-5, or IDR-1018) or the control agents (shACE2 or melittin) to neutralize betacoronavirus spike-mediated entry was determined as described by Crawford et al (63) with minor modifications. Two variations were followed:

1. *Peptides plus pseudotyped virus, then cells:* HEK293T-hACE2 or HEK293-hDPP4 cells were seeded in poly-D-lysine coated, white-walled, 96-well plates (20,000 cells/well) and incubated at 37°C, 5% CO_2_ for 24 h. Serial dilutions of the synthetic peptides or control agents were mixed with a suspension of the betacoronavirus spike-pseudotyped lentiviral particles (enough to generate >1,000-fold signal over background, approximately 3 to 4 × 10^5^ relative light units [RLU]/well) in 96-well plates at a 1:1 ratio (150 µl final volume) and incubated at 37°C, 5% CO_2_ for 1 h. The cell culture media was removed, replaced with the mixture of peptides or control agents with betacoronavirus spike pseudotyped lentiviruses, supplemented with 5 µg/mL of polybrene (Sigma-Aldrich Merck), and incubated at 37°C, 5% CO_2_ for 72 h.
2. *Peptides plus cells, then virus:* HEK293T-hACE2 or HEK293-hDPP4 cells were seeded in poly-D-lysine coated, white-walled, 96-well plates (20,000 cells/well) and incubated at 37°C, 5% CO_2_ for 24 h. Serial dilutions of the synthetic peptides or control agents were added to the cells and incubated at 37°C, 5% CO_2_ for 1 h. A suspension of the betacoronavirus spike pseudotyped lentiviral particles (enough to generate >1,000-fold signal over background, approximately 3 to 4 × 10^5^ relative light units [RLU]/well) was then added to the cells, in the presence of the synthetic peptides or control agents supplemented with 5 µg/mL of polybrene (Sigma-Aldrich Merck), and incubated at 37°C, 5% CO_2_ for 1 h. The cell culture medium was removed, replaced with fresh cell culture medium, and incubated at 37°C, 5% CO_2_ for 72 h.

In both approaches, viral entry was quantified by removing the cell culture supernatant and adding a 1:1 mixture of fresh cell culture medium (50 µL) and luciferin reagent (50 µL, Steady-Luc Firefly assay kit, Biotium, Fremont, CA) to each well. Plates were incubated at room temperature with gentle shaking (300 rpm) for 5 min, and luminescence was measured using a plate reader (VICTOR Nivo, PerkinElmer, Waltham, MA). Neutralizing antibody titers were calculated by a non-linear regression model (log inhibitor vs. normalized response-variable slope) analysis and expressed as 50% neutralizing titer (NT_50_) using GraphPad Prism v.9.2.0 (GraphPad Software, La Jolla, CA, USA).

### SARS-CoV-2 isolation

Four SARS-CoV-2 strains were isolated from patient-derived samples as described (56). Briefly, nasopharyngeal swabs were obtained from individuals with clinical signs or symptoms of COVID-19 in New Zealand between May and June 2022. Clinical specimens were transported to the laboratory in universal transport medium (UTM), where they were mixed 1:1 with high glucose DMEM (Gibco Thermo Fisher Scientific) supplemented with 5% FBS (Cellgro Mediatech), 100 units/mL of penicillin, 100 µg/mL of streptomycin, and 100 µg/mL of gentamicin (Thermo Fisher Scientific). The mixture was passed through a 0.45 µm steriflip filter (Merck Millipore, Burlington, MA) and used to inoculate VeroE6/TMPRSS2 cells (1 × 10^6^ cells/well) in a 48-well plate (Greiner Bio-One, Kremsmünster, Austria). Cells were monitored daily for cytopathic effect (CPE) for 5 days, and cell-free supernatant from positive cell cultures was used to inoculate 3 × 10^6^ VeroE6/TMPRSS2 cells in a T-25 flask (Thermo Fisher Scientific). These initial viral stocks (first serial passage or C1) were titrated by determining tissue culture dose for 50% infectivity (TCID_50_) in triplicate with CPE as the end-point using the Reed and Muench method (57). SARS-CoV-2 titers were expressed as TCID_50_ per milliliter (TCID_50_/mL).

### Whole genome sequencing of SARS-CoV-2

The four new SARS-CoV-2 isolates were sequenced using the MinION Oxford Nanopore Technologies as described (62). Briefly, the total RNA was extracted from the four C1 viral stocks (QIAamp Viral RNA mini Kit, QIAGEN, Hilden, Germany), and random nonamer primers were used to synthesize complementary DNA (cDNA), and Sequenase Version 2.0 DNA Polymerase (Thermo Fisher Scientific) was used to generate second-strand DNA. cDNA/second-strand products were amplified, PCR products were purified (QIAquick PCR Purification kit, Qiagen), and the concentration of double-stranded cDNA was quantified (Qubit 2.0, Thermo Fisher Scientific). A pool of purified PCR products, averaging ~700 bp (Qubit dsDNA HS assay kit, Thermo Fisher Scientific), was diluted to a starting concentration of 200 fmol following the recommended protocol (NBA_9093_v109_12Nov2019, Oxford Nanopore Technologies), processed using the NEBNext Ultra II End Repair/dA-Tailing Module (New England BioLabs, USA), and barcode adapters (Native Barcoding Expansion 1-12 EXP-NBD104 kit, Oxford Nanopore Technologies). were added to the repaired amplicons using the Ligation Sequencing Kit (SQK-LSK109, Oxford Nanopore Technologies), followed by DNA purification (Agencourt AMPure XP, Beckman Coulter). Individually barcoded DNA samples were quantified (Qubit 2.0, Thermo Fisher Scientific) and mixed at equimolar concentrations. The pooled DNA library was loaded onto a primed FLO-MIN106 MinION R9.4.1 flow cell (Oxford Nanopore Technologies) and run for 24 h. MinKNOW Core 3.6.5 (Oxford Nanopore Technologies) was run on the MinIT device (Oxford Nanopore Technologies) for real-time analysis, basecalling, and barcode demultiplexing and to generate sample-specific fastq files. Finally, FASTQ files were analyzed using a combination of software packages to characterize the whole genome SARS-CoV-2 sequences: (i) GISAID (https://www.gisaid.org/), (ii) Genome Detective Virus Tool (https://www.genomedetective.com/), (iii) Stanford University Coronavirus Antiviral & Resistance Database (https://covdb.stanford.edu/), and (iv) CZ ID (https://czid.org/).

### Phylogenetic analysis

A set of 21 whole genome SARS-CoV-2 sequences, representative of viruses considered variants of concern (VOC) by the World Health Organization, was downloaded on 30 June 2022 from the GISAID database (https://www.gisaid.org) (64) to assess the phylogeny of the SARS-CoV-2 sequences described in this study. Whole genome SARS-CoV-2 sequences were aligned using ClustalW (65), and their phylogeny was reconstructed using the maximum likelihood model with bootstrap as the variance estimation method (1,000 replicates) implemented within MEGA 6.1 (66).

### Inhibition of hACE2-SARS-CoV-2 RBD binding assay

A competitive enzyme-linked immunosorbent assay (ELISA) was used to test the ability of the synthetic peptides or control agents to inhibit the binding between RBD-Fc and hACE2-alkaline phosphatase (AP) fusions. Briefly, 50 µL of a solution at 3 µg/mL of mouse anti-AP (PLAP Ab-1 [8B6.18] Mouse Mab; NeoMarkers, Portsmouth, NH, USA) in 1× PBS was added to each well of a 96-well plate using an automated multichannel pipette, sealed, and incubated overnight at 4°C. The following day, the plates were washed with PBS and incubated with 5% non-fat dry milk in PBS (as a blocking agent) for 1 h at room temperature. The blocking agent was then removed, by inverting the plate, and 20 µL of the ecto-AP conditioned medium containing 2 µL of monoclonal mouse anti-human IgG1-HRP (2 µg/mL, AbD Serotec Bio-Rad, Oxford, United Kingdom) was added to each well using an automated plate copier (VIAFLO 96/384 Integra, Princeton, MA, USA) along with 20 µL of ecto-Fc culture medium. Plates were sealed and incubated for 4 h at room temperature, washed (3×) with 400 µL of wash buffer containing 1× Tris-Buffered saline and 0.1% Tween. To each well, 50 µL/well of 1-Step Ultra TMB-ELISA Horseradish peroxidase (HRP) substrate solution (ThermoFisher Scientific) was added and incubated for 15 min at room temperature, prior to measuring absorbance at 650 nm using a microplate reader to detect the binding between hACE2-AP and SARS-CoV-2 RBD-Fc. Finally, the 96-well plates were scanned to record matching images of the absorbance readings. Positive controls (shACE2 and SARS-CoV-2 RBD) were used to gauge the sensitivity of the assay, and negative controls (wells with either no anti-AP antibody or no anti-human Fc-HRP) were used to obtain background values to normalize the positive reactions.

### Drug combination dose-response analysis

The effect of the combination of the synthetic peptide D-3006 with shACE2 or remdesivir was assessed using both the SARS-CoV-2 (Wuhan) spike-pseudotyped virus neutralization assay and the replication-competent SARS-CoV-2 hCoV-19/New Zealand/NZ1_patient/2020 strain, as described above. Briefly, based on a constant ratio combination design, the EC_50_ values were determined for each compound alone and in combination at the following concentrations: 0, 1, 5, and 10 µg/mL (D-3006 and shACE2) or µM (remdesivir). The degree of synergy, additivity, or antagonistic effect was quantified using the SynergyFinder web application v2.0 (67), based on the Zero Interaction Potency (ZIP) reference model that assumes no interaction between drugs. A four-parameter logistic regression model was used to fit the inhibition curves. Synergy scores for the drug combinations were calculated and visualized as heat maps and 3D synergy maps. Synergy scores were interpreted as the average excess due to drug interactions, that is, a synergy score of 15 for a given drug combination corresponded to a dose effect 15% higher than expected for either drug alone. Synergy scores below −10, between −10 and 10, or above 10 suggested that interaction between the two drugs was likely to be antagonistic, additive, or synergistic, respectively (67).

### Drug susceptibility assay using replication-competent influenza A virus

The susceptibility of IAV A/Mallard/Alberta/287/2012 (H1N1) to D-3006 and favipiravir (FVP, T-705, Sapphire Bioscience, Waterloo, Australia) was evaluated in MDCK or ELVIRA Flu A cells. Serial dilutions spanning empirically determined ranges of the synthetic peptide or FPV were added in triplicate to 96-well plates containing MDCK or ELVIRA Flu A cells (20,000 cells/well) and incubated at 37°C, 5% CO_2_ for 2 h. Cells were then infected with IAV at a MOI of 0.01 IU/cell for 1 h at 37°C, 5% CO_2_. Virus inoculum was removed, cells were washed twice, and complete medium with the corresponding agent dilution was replenished. IAV replication was quantified 72 h post-infection by (i) directly quantifying the expression of the firefly luciferase gene in ELVIRA Flu A cells in response to infection with IAV (55) or (ii) in a virus-yield assay, by collecting the virus in the supernatant of MDCK cells and using ELVIRA Flu A cells as reporter cells. Concentrations of D-3006 or FVP required to inhibit IAV replication by 50% (EC_50_) were calculated by plotting the percent inhibition of virus replication versus log_10_ drug concentration and fitting the inhibition curves to the data using nonlinear regression analysis (GraphPad Prism v.9.3.1, GraphPad Software).

### Electron microscopy

SARS-CoV-2 (NZ1) or IAV (H1N1) stocks were grown in VeroE6/TMPRSS2 or MDCK cells, respectively; cell-culture supernatant was harvested, clarified by centrifugation at 1,500 × *g* rpm for 10 min, and filtered through a 0.45-µm steriflip filter (Merck Millipore, Burlington, MA, USA). Four milliliter virus aliquots were treated with D-3006, melittin, or 1× DMEM for 1 h at 37°C and 5% CO_2_, prior to fixing them with 2% formaldehyde to inactivate the viruses. Fixed virus aliquots were removed from the BSL-3 laboratory and ultracentrifuged at 80,000 × *g* RCF for 90 min through a 20% sucrose cushion and resuspended in 1× PBS. Five microliter aliquots of each sample were incubated for 1 min on plasma glow-discharged carbon-coated copper grids (Electron Microscopy Sciences, Hatfield, PA, USA), blotted and negatively stained with 10  µL of 1% phosphotungstic acid, blotted, and then air-dried. Grids were investigated using a Philips CM100 transmission electron microscope (TEM) at the Otago Micro and Nanoscale Imaging (OMNI, University of Otago, New Zealand) with an accelerating voltage of 100 KeV. To quantify and qualify the structure of the virus particles, the micrographs were obtained at 46,000× from 15 random fields across three grid cells.

### Statistical analysis

Descriptive results are expressed as median values, standard deviations, and confidence intervals. Group medians were compared using a two-sided Wilcoxon-Mann-Whitney test. The cutoff level for significance was set at 0.05 (*P* < 0.05). All statistical analyses were performed using GraphPad Prism v.9.2.0 (GraphPad Software, USA) unless otherwise specified.

## RESULTS

### Synthetic host defense peptides were not cytotoxic and inhibited SARS-CoV-2 replication *in vitro*

**Fig 1:**
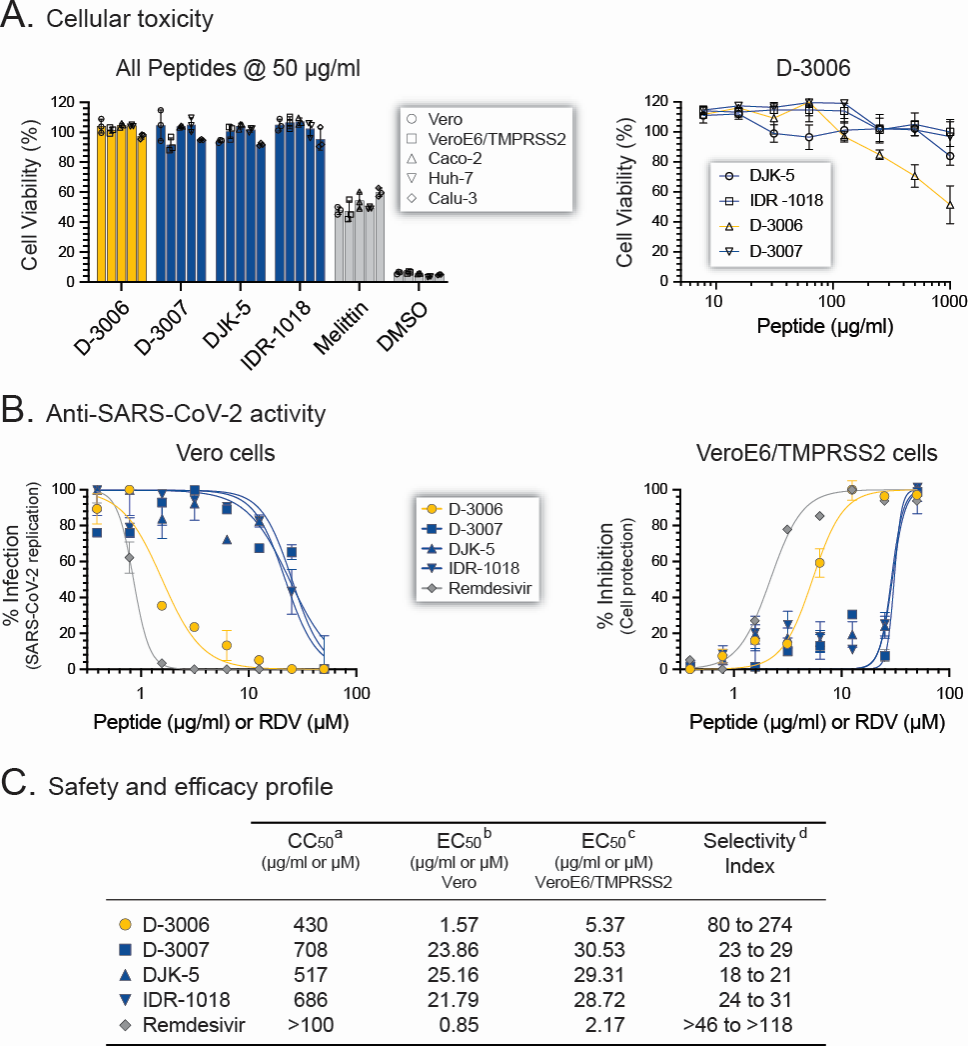
Cellular toxicity and anti-SARS-CoV-2 activity of synthetic host defense peptides. (**A**) The viability of Vero, VeroE6/TMPRSS2, Caco-2, Huh-7, and Calu-3 cells exposed to 50 µg/mL of D-3006, D-3007, DJK-5, or IDR-1016, as well as melittin or DMSO as controls, was determined in triplicate (left panel). The dose-dependent cytotoxic effect was calculated for D-3006 in VeroE6/TMPRSS2 cells in triplicate (right panel), with the cell viability percentage fitted by a four-parameter log-logistic model to determine half-maximum (50%) cytotoxic concentration (CC_50_) values. (**B**) Susceptibility of the authentic SARS-CoV-2 hCoV-19/New Zealand/NZ1_patient/2020 isolate (56) to the four synthetic host defense peptides (D-3006, D-3007, DJK-5, or IDR-1018) and remdesivir in Vero or VeroE6/TMPRSS2 cells. SARS-CoV-2 replication was quantified 72 h post-infection by CPE, RT-qPCR assay (62), or a cell protection assay based on the Pierce BCA protein assay kit (Thermo Fisher Scientific). EC_50_ values, determined using the non-linear regression model (log inhibitor vs. normalized response-variable slope), are depicted in µg/mL for the synthetic peptides or µM for remdesivir. (**C**) *In vitro* safety and efficacy (CC_50_ and EC_50_ values, respectively) profiles, calculated in Vero and VeroE6/TMRPSS2 cells, were used to determine the selectivity index (CC_50_/EC_50_) for each synthetic host defense peptide and remdesivir.

Characterization of any potential antiviral agent must start by assessing its effect on cellular metabolism and survival (8). Here, we first quantified the effect of four synthetic peptides, that is, D-3006, D-3007, DJK-5, or IDR-1018, in cell viability and proliferation. Limited cytotoxicity was observed when Vero and VeroE6/TMPRSS2 cells, two kidney epithelial cell lines derived from an African green monkey and commonly used to isolate and propagate viruses such as SARS-CoV-2 (56), were exposed to 50 µg/mL of the synthetic peptides. Similar results were observed when this relatively high concentration of the synthetic peptides was used with three different epithelial-like cell lines obtained from human colon (Caco-2), liver (Huh-7), or lung (Calu-3) tissues (Fig. 1A). As expected, 50 µg/mL of the basic peptide melittin, which causes pore-formation in cell membranes (68), or 5% dimethyl sulfoxide (DMSO) known to induce cell death (69), reduced viability in all cell lines (mean cell viability 51.7% and 5.4%, respectively, Fig. 1A). We then used serial dilutions of D-3006 (Fig. S1) and the other three synthetic peptides (D-3007, DJK-5, and IDR-1018, data not shown) showing non-detectable cellular toxicity in all five cell lines for all four peptides at concentrations as high as 100 µg/mL. Finally, we determined the 50% cytotoxic concentration (CC_50_) values of the four synthetic peptides in VeroE6/TMPRSS2 cells (the cell line used to evaluate antiviral activity), which ranged from 430 µg/mL for D-3006 to 708 µg/mL for D-3007 (Fig. 1A and C).

We tested the anti-SARS-CoV-2 activity of the four synthetic peptides using a replication-competent (authentic) SARS-CoV-2 isolate (hCoV-19/New Zealand/NZ1_patient/2020) in Vero cells. D-3007, DJK-5, and IDR-1018 inhibited SARS-CoV-2 replication with EC_50_ values ranging from 21.8 to 25.2 µg/mL; however, D-3006 was 14-fold more potent, blocking SARS-CoV-2 replication with an EC_50_ value of 1.57 µg/mL (Fig. 1B and C). A similar profile was obtained when the four synthetic peptides were tested using the most permissive VeroE6/TMPRSS2 cells (54, 70), that is, EC_50_ values for D-3007, DJK-5, and IDR-1018 ranged from 28.7 to 30.5 µg/mL, whereas D-3006 inhibited SARS-CoV-2 replication with an EC_50_ value of 5.37 µg/mL (Fig. 1B and C). Based on these results, we were able to determine selectivity index (SI) values for each peptide. Although D-3007, DJK-5, and IDR-1018 showed modest SI values, ranging from 18 to 31, the selectivity indexes in both Vero and VeroE6/TMPRSS2 cells for D-3006 were closer to the SI values determined for remdesivir, that is, ranging from 80 to 274 cf. >46 to >118, respectively (Fig. 1C).

### Production and characterization of soluble human ACE2

As described above, as a control in our original antiviral assay, we used remdesivir, an FDA-approved nucleoside analog that inhibits the RNA-dependent RNA polymerase (RdRp) of SARS-CoV-2 and other RNA viruses (71). Since we needed a control molecule that would block SARS-CoV-2 entry, particularly the interaction of the virus with its cellular receptor (72), we used both a commercial SARS-CoV-2 spike-neutralizing monoclonal antibody and a soluble version of human ACE2 (shACE2). For that, we constructed and fully characterized a secreted dimeric version of hACE2, produced by a stable cell line expressing the extracellular domain of the protein (Fig. S2A and B). We then verified the expression of shACE2 using western blot and tested the ability of the purified protein (upon cleavage of the human Fc portion) to bind to the SARS-CoV-2 RBD (Fig. S2C). As expected, the purified shACE2 inhibited the interaction of hACE2-AP to RBD-Fc (SARS-CoV-2 Wuhan strain) with an IC_50_ of 1.42 µg/mL (Fig. S2D). Finally, we tested the ability of our shACE2 protein to block SARS-CoV-2 entry, using a SARS-CoV-2 (Wuhan) spike-pseudotyped virus and the authentic SARS-CoV-2 isolate hCoV-19/New Zealand/NZ1_patient/2020. As shown in Fig. S2E, both the commercial SARS-CoV-2 spike-neutralizing monoclonal antibody and the shACE2 protein inhibited entry of the SARS-CoV-2 spike pseudotyped virus into HEK293T-hACE2 cells (EC_50_ values of 0.014 µg/mL and 0.93 µg/mL, respectively) and blocked the replication of the authentic SARS-CoV-2 in VeroE6/TMPRSS2 cells (EC_50_ values of 0.028 µg/mL and 3.32 µg/mL, respectively).

**Fig 2:**
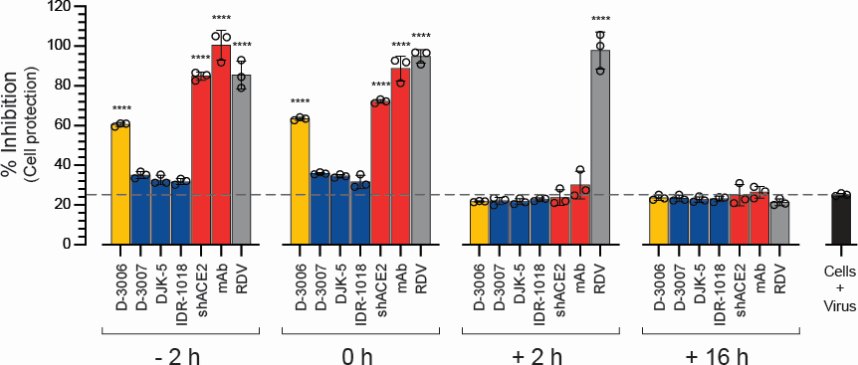
Time-of-drug addition analysis of synthetic host defense peptides. VeroE6/TMPRSS2 cells were infected with authentic SARS-CoV-2 hCoV-19/New Zealand/NZ1_patient/2020 in the presence and absence of the four synthetic peptides (D-3006, D-3007, DJK-5, or IDR-1018) or the control agents (shACE2, RDV, or a SARS-CoV-2 spike neutralizing antibody mAb) added 2 h prior (−2 h), during (0 h), or 2 h (+2 h) or 16 h (+16 h) after infection at a concentration equal to 10-fold of their respective EC_50_ values (as described in Fig. 1). Inhibition of SARS-CoV-2 replication was quantified 72 h post-infection using a cell protection assay based on the Pierce BCA protein assay kit (Thermo Fisher Scientific) (56). Cells + virus, SARS-CoV-2 replication in the absence of inhibitory agent. Depicted values represent medians ± standard deviations (SD) from three replicates. Wilcoxon-Mann-Whitney test was used to compare the % inhibition of SARS-CoV-2 replication between the different conditions and the Cells + virus control. **** *P* < 0.0001.

### The host defense peptide D-3006 blocked SARS-CoV-2 entry

To get a first glimpse of the potential mechanism of action of the synthetic host defense peptides, we first used a time-of-drug addition assay to compare the inhibition profile of the novel peptides with those of well-known anti-SARS-CoV-2 agents. We infected VeroE6/TMPRSS2 cells with the authentic SARS-CoV-2 hCoV-19/New Zealand/NZ1_patient/2020 strain and added the synthetic host defense peptides or the control agents (shACE2, SARS-CoV-2 spike-neutralizing antibody, and RDV), at selected time points (Fig. 2). The shACE2 and the SARS-CoV-2 spike-neutralizing antibody were able to block SARS-CoV-2 replication in their expected temporal order, that is, blocking virus replication when added simultaneously (72% and 89% inhibition, respectively) or 2 h before the infection of the cells with SARS-CoV-2 (85% and 100% inhibition, respectively). On the other hand, the RdRp inhibitor RDV inhibited SARS-CoV-2 even when added 2 h post-infection (98% inhibition, Fig. 2). Interestingly, all four synthetic host defense peptides showed an inhibitory profile similar to that of the entry inhibitors, that is, shACE2 and SARS-CoV-2 spike-neutralizing antibody. Although D-3007, DJK-5, and IDR-1018 showed relatively limited inhibition of SARS-CoV-2 replication (mean inhibition of 35%, 33%, and 31%, respectively), D-3006 was more efficient at reducing virus replication when added together or 2 h before SARS-CoV-2 infection (60% and 63% inhibition, respectively, Fig. 2).

**Fig 3:**
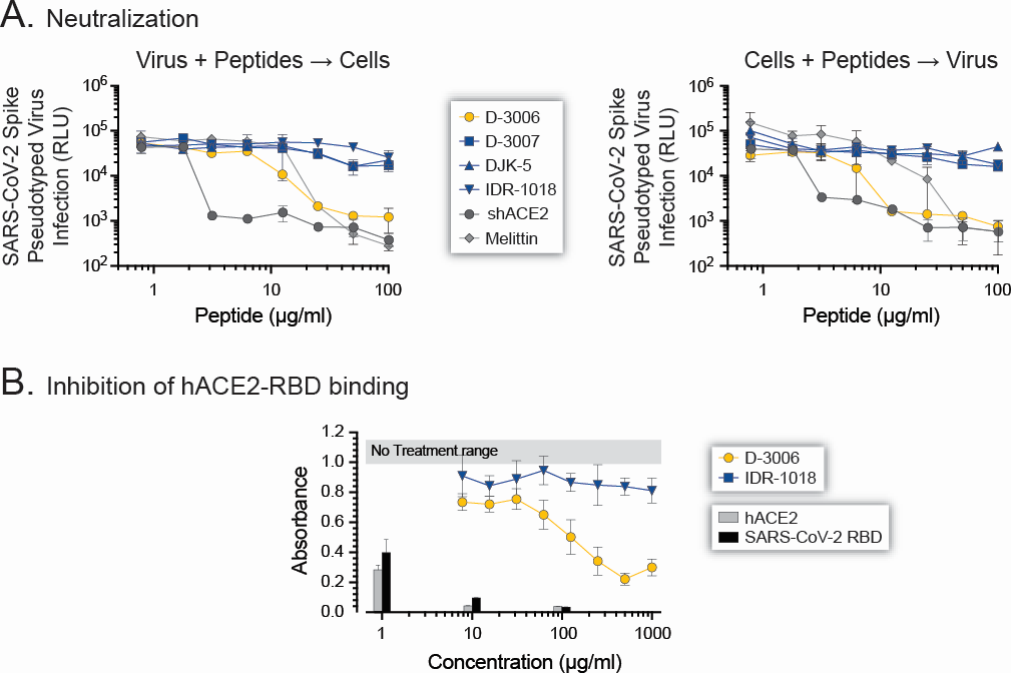
D-3006 neutralized SARS-CoV-2 spike-pseudotyped viruses. (**A**) Synthetic host defense peptides (D-3006, D-3007, DJK-5, or IDR-1018) or control agents (shACE2 or melittin) were incubated with the SARS-CoV-2 (Wuhan) spike-pseudotyped virus (left panel) or with HEK293T-hACE2 cells (right panel) for 1 h prior to adding the cells or pseudotyped virus, respectively. SARS-CoV-2 spike-mediated entry was quantified 72 h post-infection, and neutralizing titers (50% neutralizing titer, NT_50_) were determined. (**B**) Dose-dependent inhibition of hACE2-RBD binding by the synthetic peptides D-3006 and IDR-1018, as well as soluble hACE2 and SARS-CoV-2 RBD as controls, using a competitive ELISA as described in Fig. S2. Absorbance was determined at 650 nm. Depicted values represent medians ± standard deviations (SD) from three replicates. The horizontal gray bar represents the no treatment control, ranging from 1 to 1.18.

Based on the time-dependent activity profile of the synthetic host defense peptides, we tested their ability to block viral entry using a SARS-CoV-2 spike-pseudotyped virus system based on the ancestral Wuhan strain (63). We first evaluated neutralization activity by incubating SARS-CoV-2 Wuhan spike-pseudotyped virus particles with increasing concentrations of the synthetic host defense peptides or the control agents (shACE2 and melittin). Similar to the results obtained with the authentic SARS-CoV-2 in Vero and VeroE6/TMPRSS2 cells (Fig. 1B), as well as the time-of-drug-addition assay (Fig. 2), D-3007, DJK-5, and IDR-1018 failed to significantly neutralize the SARS-CoV-2 Wuhan spike-pseudotyped virus (Fig. 3A). On the other hand, D-3006 was able to block the ability of the SARS-CoV-2 Wuhan spike-pseudotyped virus to enter HEK293T-ACE2 cells with an NT_50_ value of 5.63 µg/mL, better than the non-specific bee venom peptide melittin (NT_50_ of 12.5 µg/mL) and only 2-fold less potent than the highly specific shACE2 (NT_50_ of 2.81 µg/mL, Fig. 3A). We observed similar results when the HEK293T-ACE2 cells were incubated with the antiviral agents, prior to being exposed to the SARS-CoV-2 Wuhan spike-pseudotyped virus, that is, limited viral entry inhibition with D-3007, DJK-5, and IDR-1018, whereas D-3006, melittin, and shACE2 blocked entry of the SARS-CoV-2 Wuhan spike-pseudotyped virus with EC_50_ values of 5.84, 13.2, and 2.38 µg/mL, respectively (Fig. 3A).

We then used a competitive ELISA to test the ability of two of the synthetic host defense peptides, that is, the most potent D-3006 and the less efficient IDR-1018, to block binding of the SARS-CoV-2 Wuhan RBD to the hACE2 receptor. As shown in Fig. 3B, 1,000 µg/mL of IDR-1018 failed to inhibit binding of SARS-CoV-2 RBD-Fc to the hACE2-AP. Interestingly, although less potent than the hACE2 and SARS-CoV-2 RBD controls (IC_50_ values of 6.6 and 8.6 µg/mL, respectively), D-3006 was able to outcompete RBD-Fc, blocking the binding to hACE2-AP with an IC_50_ of 131.3 µg/mL (Fig. 3B). Finally, we verified the potential ability of D-3006 to block viral entry by first quantifying the antiviral activity of the HDP using the SARS-CoV-2-Nluc strain in VeroE6/TMPRSS2 cells, obtaining an EC_50_ value (5.4 µg/mL, Fig. S3A) similar to the one determined with the authentic SARS-CoV-2 hCoV-19/New Zealand/NZ1_patient/2020 strain (5.37 µg/mL, Fig. 1). Interestingly, 100 µg/mL of D-3006 completely inhibited the ability of the SARS-CoV-2-mNG strain (expressing mNeonGreen) to enter VeroE6/TMPRSS2 cells (Fig. S3B).

### *In vitro* synergism of D-3006 and remdesivir against SARS-CoV-2

**Fig 4:**
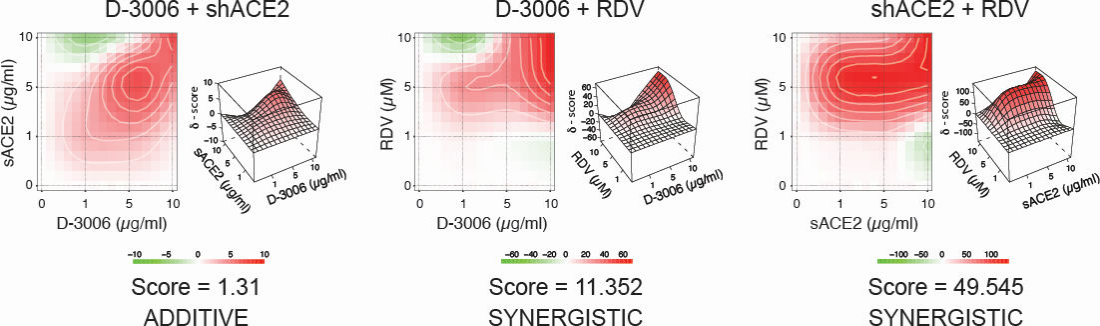
Combination treatment of D-3006 with shACE2 or remdesivir. VeroE6/TRMPSS2 cells were infected with authentic SARS-CoV-2 (hCoV-19/New Zealand/NZ1_patient/2020 strain) in the presence and absence of each agent alone and in combination at the following concentrations: 0, 1, 5, and 10 µg/mL (D-3006 and shACE2) or µM (remdesivir), and the degree of synergistic, additivity, or antagonistic effect quantified using SynergyFinder (67). Scores for drug combinations were visualized as heatmaps and 3D synergy maps and interpreted as the average excess due to drug interactions. Scores < −10, in the range of −10 to 10, or >10 suggest that interaction between the two agents is likely to be antagonistic, additive, or synergistic, respectively (67).

Since the synthetic host defense peptide D-3006 inhibited entry of both authentic SARS-CoV-2 and SARS-CoV-2 spike-pseudotyped viruses, we tested its antiviral activity in two-drug combinations with the entry inhibitor shACE2 or the RdRp inhibitor RDV. HEK293T/hACE2 cells exposed to combinations of D-3006 with shACE2 showed an additive effect blocking entry of the SARS-CoV-2 (Wuhan) spike-pseudotyped virus with a score of −1.902 (Fig. S4). Not surprisingly, combinations of D-3006 or shACE2 with RDV also showed an additive effect blocking entry of the SARS-CoV-2 spike pseudotyped virus into HEK293T/hACE2 cells (scores of −0.772 and −2.371, respectively, Fig. S4), mainly because RDV blocks viral replication at a post-entry step of the viral cycle, not measured in a pseudotyped virus system. A similar additive effect was observed when the combination of D-3006 and shACE2 was used to inhibit the infection of VeroE6/TMPRSS2 cells with the authentic SARS-CoV-2 (synergy score of 1.31, Fig. 4). Interestingly, the combination of D-3006 with RDV showed a synergistic effect, potently blocking replication of the authentic SARS-CoV-2 with a synergistic score of 11.352, similar to the activity obtained with the shACE2 plus RDV combination (synergist score = 49.545, Fig. 4).

### Pan-β-coronavirus activity of D-3006

**Fig 5:**
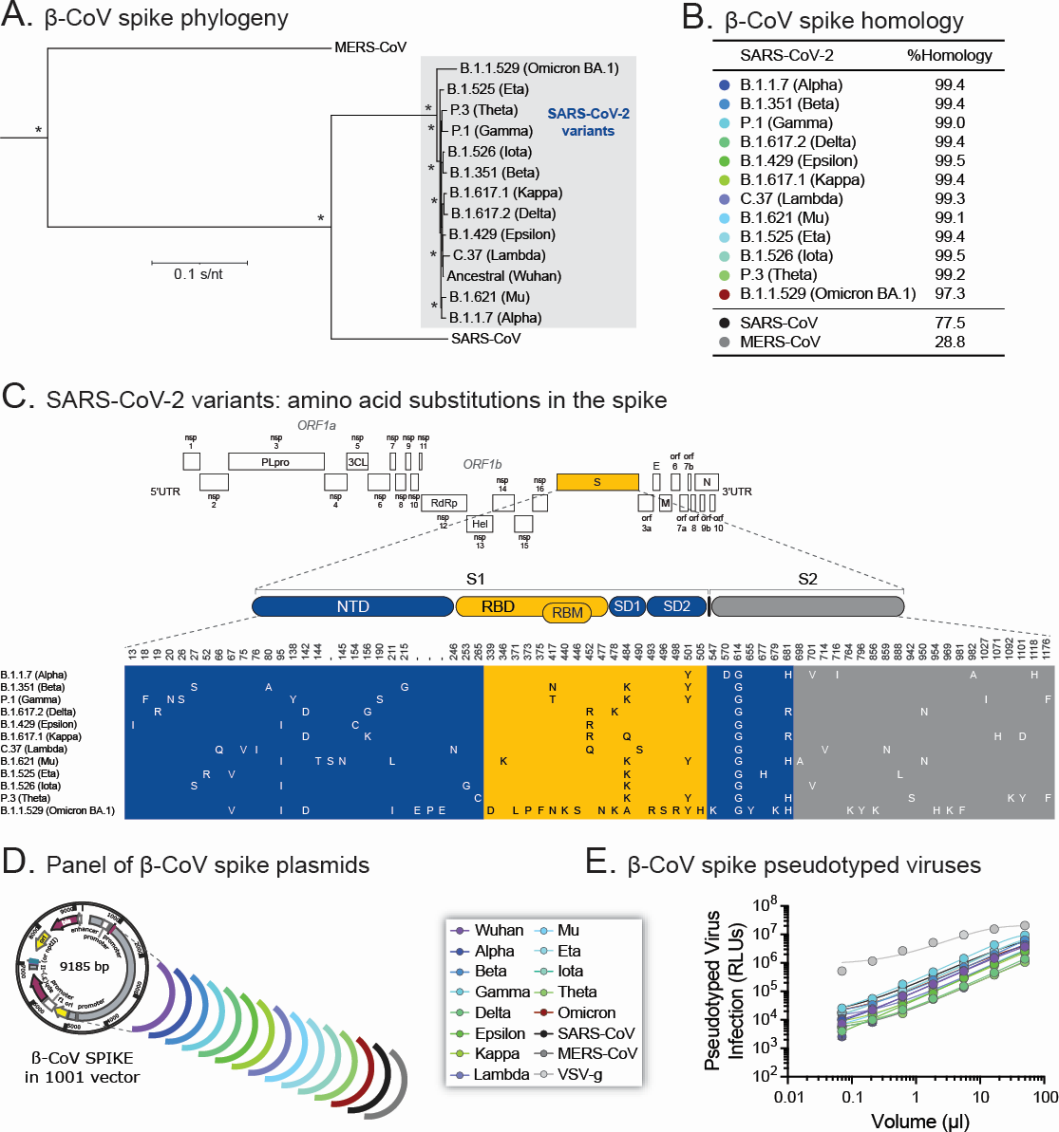
Generation of a comprehensive panel of betacoronavirus (β-CoV) spike-pseudotyped viruses. (**A**) Spike protein sequences from 13 SARS-CoV-2 variants from the ancestral Wuhan to Omicron BA.1, together with SARS-CoV and MERS-CoV, were aligned, and a maximum likelihood phylogenetic tree was constructed. Bootstrap resampling (1,000 data sets) of the multiple amino acid alignment tested the statistical robustness of the tree, with percentage values above 75% indicated by an asterisk. SARS-CoV-2 variants are enclosed in a gray box. S/nt, substitutions per nucleotide. (**B**) A pairwise comparison analysis of spike protein sequence identities, quantified as % homology, was used to determine the relatedness of the 14 betacoronavirus spike protein sequences with that of the SARS-CoV-2 Wuhan strain. (**C**) Schema of the SARS-CoV-2 Wuhan genome listing the amino acid substitutions of the 12 SARS-CoV-2 variants of concern (Alpha to Omicron BA.1) in the spike (S) gene compared with the reference Wuhan strain. S1 and S2 spike regions; NTD, N-terminal domain; RBD, receptor-binding domain; RBM, receptor-binding motif; SD1 and SD2, spike subdomains 1 and 2, respectively. (**D**) Schema of the 16 viral proteins, 15 betacoronavirus spikes, and one VSV-g, cloned into the 1001 vector (a modified pcDNA3.1 plasmid) to generate pseudotyped lentiviruses. (**E**) Viral titers (TCID_50_ values) were determined using serial dilutions of the betacoronavirus spike or VSV-g pseudotyped lentiviruses to infect HEK293T-hACE2 cells (for all SARS-CoV-2 variants, SARS-CoV, and VSV-g) or HEK293-hDPP4 cells (for MERS-CoV). RLUs, relative light units.

Since the synthetic host defense peptide D-3006 was able to (i) inhibit the replication of the authentic SARS-CoV-2 hCoV-19/New Zealand/NZ1_patient/2020 in Vero and VeroE6/TMPRSS2 cells, (ii) block the entry of a SARS-CoV-2 spike pseudotyped virus based on the ancestral Wuhan strain into HEK293T-ACE2 cells, and (iii) show synergism with RDV in both authentic and SARS-CoV-2 spike-pseudotyped virus systems, we next evaluated the ability of D-3006 to inhibit spike-mediated entry of other betacoronaviruses. For that, we first constructed and characterized a panel of 15 β-coronavirus spike pseudotyped viruses including all 13 original SARS-CoV-2 variants of concern (from Alpha to Omicron BA.1), as well as the more diverse SARS-CoV and MERS-CoV (Fig. 5A and B). Most of the genetic diversity of SARS-CoV-2 variants is associated with amino acid substitutions in the spike glycoprotein (Fig. 5C), representing the adaptation of the novel coronavirus to its new human host (73, 74). These β-coronavirus spikes were synthesized and cloned into a modified pcDNA3.1 vector (Fig. 5D) to generate a complete set of β-coronavirus spike-pseudotyped viruses, including a virus containing the VSV-g envelope protein as control (Fig. 5E).

**Fig 6:**
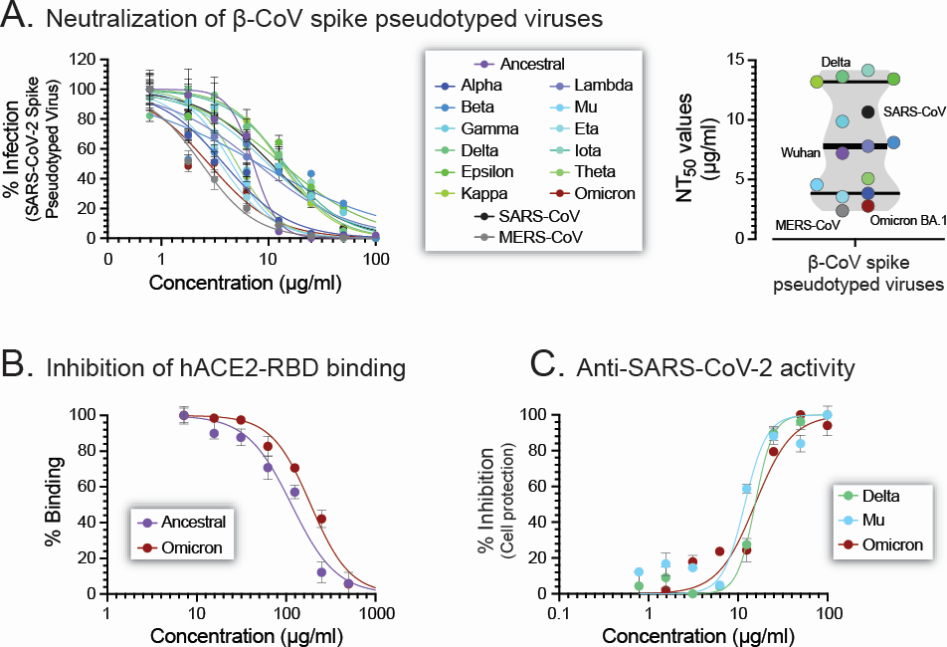
Broad neutralization activity of D-3006. (**A**) The synthetic host defense peptide neutralized lentiviruses pseudotyped with spike proteins from 13 SARS-CoV-2 strains (ancestral/Wuhan to Omicron BA.1), SARS-CoV, and MERS-CoV. See Fig. 4 and 5 legends, as well as Materials and Methods, for details. (**B**) Dose-dependent inhibition of hACE2-RBD binding by D-3006 using a competitive ELISA based on the RBD protein sequence from the ancestral (Wuhan) and Omicron (BA.1). (**C**) Susceptibility of the authentic SARS-CoV-2 Delta, Mu, and Omicron BA.1 strains to D-3006 in VeroE6/TMPRSS2 cells as described in Fig. S2. Depicted values represent medians ± standard deviations (SD) from three replicates. NT_50_, 50% neutralizing titer.

Using this panel of β-coronavirus spike-pseudotyped viruses, we showed that D-3006 was able to neutralize not only all 13 SARS-CoV-2 spike-pseudotyped viruses (NT_50_ values ranging from 2.78 to 14.2 µg/mL) but also the more diverse SARS-CoV (10.7 µg/mL) and MERS-CoV (2.38 µg/mL) spike-pseudotyped viruses (Fig. 6A). In addition, we used a competitive ELISA to verify the ability of D-3006 to inhibit the binding of SARS-CoV-2 Wuhan RBD (IC_50_ of 115.2 µg/mL), as well as SARS-CoV-2 Omicron BA.1 RBD (IC_50_ of 189 µg/mL), to the hACE2 receptor (Fig. 6B). Finally, we expanded the results obtained with the authentic SARS-CoV-2 ancestral (Wuhan) strain (Fig. 1B and C) by first isolating and characterizing additional SARS-CoV-2 variants circulating in New Zealand in June 2022, that is, delta (δ), mu (μ), and omicron (ο) BA.1 (Fig. S5). D-3006 inhibited the replication of the three authentic SARS-CoV-2 variants, that is, SARS-CoV-2 δ (hCoV-19/New Zealand/NZ8_patient/2022), μ (hCoV-19/New Zealand/NZ10_patient/2022), and ο BA.1 (hCoV-19/New Zealand/NZ11_patient/2022) in VeroE6/TMPRSS2 cells with EC_50_ values of 15.5, 11.9, and 15.4 µg/mL, respectively (Fig. 6C).

### D-3006 did not seem to affect the integrity of viral membranes

**Fig 7:**
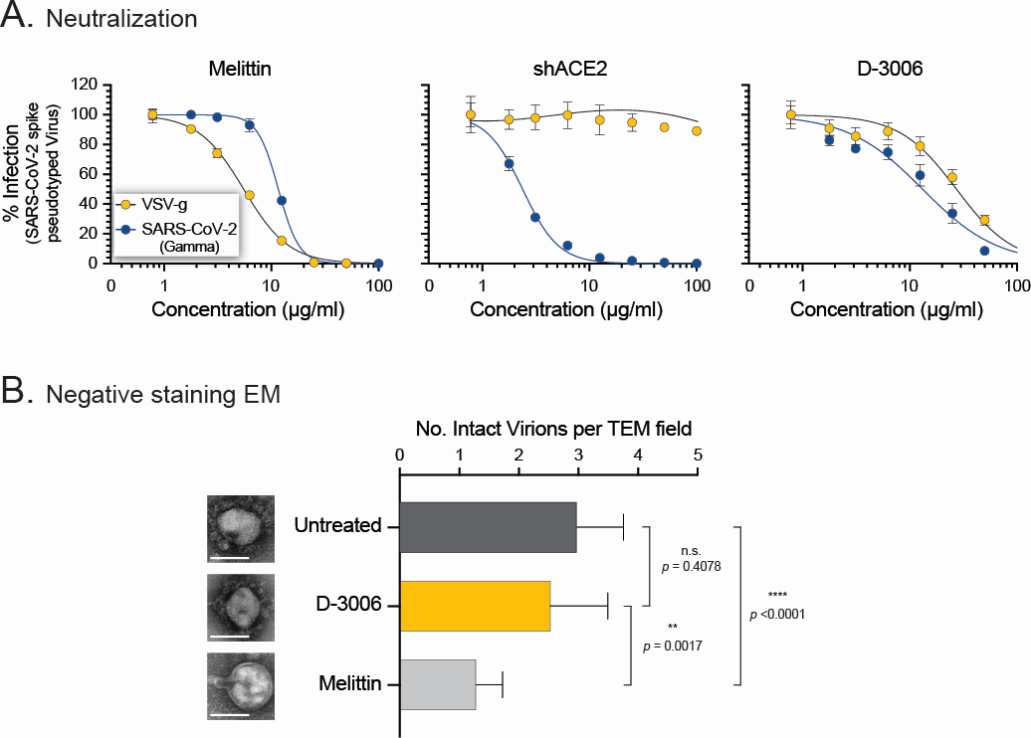
D-3006 did not show a virucidal effect. (**A**) Lentiviruses pseudotyped with the SARS-CoV-2 Gamma spike of VSV-g proteins were exposed to melittin, shACE2, or D-3006 for 1 h before adding them to HEK293T-hACE2 cells. Dose-dependent viral entry inhibition was quantified 72 h post-infection, and neutralizing titers (NT_50_) were determined. (**B**) Aliquots of SARS-CoV-2 hCoV-19/New Zealand/NZ1_patient/2020 strain were incubated with 100 µg/mL of D-3006, melittin, or 1× DMEM (Untreated) for 1 h at 37°C and 5% CO_2_, fixed with 2% formaldehyde, purified (20% sucrose cushion), and prepared for negative staining transmission electron microscopy analysis. Viral particles were evaluated and quantified in 15 random fields per condition. Single SARS-CoV-2 particles, per condition, are shown; however, additional electron microscopy images - as well as raw data - are available upon request. Depicted values represent medians ± standard deviations (SD). Wilcoxon-Mann-Whitney test was used to compare the number of intact virions per field between the untreated, D-3006, and melittin conditions. n.s., not significant.

A number of antimicrobial peptides have been shown to interact with lipid bilayers, most likely disrupting the virus membrane and contributing to their antiviral activity (41, 48). Here, we first tested the potential virucidal effect of D-3006 by incubating viruses pseudotyped with SARS-CoV-2 gamma (γ) spike glycoprotein or VSV-g envelope protein with the synthetic host defense peptide or two control agents, that is, the known virucidal melittin (68, 75) and the SARS-CoV-2 receptor-specific shACE2 (76). As expected, melittin neutralized both SARS-CoV-2 γ spike- and VSV-g-pseudotyped viruses (NT_50_ values of 11.6 and 5.51 µg/mL, respectively). On the other hand, shACE2 specifically inhibited the entry of the SARS-CoV-2 γ spike-pseudotyped virus into HEK293T-hACE2 cells (NT_50_ 2.38 µg/mL) but failed to neutralize the VSV-g-pseudotyped virus (NT_50_ >100 µg/mL). Interestingly, the synthetic host defense peptide D-3006 neutralized both SARS-CoV-2 γ spike (NT_50_ 13.1 µg/mL) and VSV-g (NT_50_ 27.3 µg/mL) pseudotyped viruses (Fig. 7A).

Since D-3006 seemed to interact with the viral and/or cell membrane and to further explore its potential virucidal effect, we incubated authentic SARS-CoV-2 δ (hCoV-19/New Zealand/NZ9_patient/2022) with 100 µg/mL of D-3006 or melittin and analyzed its effect using transmission electron microscopy (TEM). As expected, negative-stain TEM showed partial or complete disruption of the viral membrane in authentic SARS-CoV-2 virions treated with melittin, significantly reducing the number of intact virions compared with the untreated virus control (1.3 vs 3 mean number of intact virus particles per field, respectively, *P* < 0.0001, Fig. 7B). Interestingly, although slightly reduced, D-3006 did not seem to disrupt the membrane of SARS-CoV-2 virions compared with the untreated control samples (2.6 vs 3 mean number of intact virus particles per field, respectively, *P* = 0.408, Fig. 7B).

To further investigate the effect and specificity of D-3006 in viral membranes, we tested the ability of the synthetic host defense peptide to inhibit the replication of the influenza A virus. Similar to the results with Vero, VeroE6/TMPRSS2, Caco-2, Huh-7, and Calu-3 cells (Fig. 1A), 50 µg/mL of D-3006 did not affect the viability of MDCK or ELVIRA Flu A cells (Fig. S6). More importantly, 50 µg/mL of D-3006 significantly inhibited the replication of IAV A/Mallard/Alberta/287/2012 (H1N1) strain in both ELVIRA Flu A (*P* = 0.0075) and MDCK (*P* = 0.0001) cells, to levels comparable with the influenza viral RNA polymerase inhibitor favipiravir (Fig. S6). Finally, just like with the authentic SARS-CoV-2 virus, and unlike melittin, negative-stain TEM analysis showed that D-3006 did not affect the integrity of the membrane of the IAV virions (Fig. S7).

## DISCUSSION

Myriads of antivirals have been tested over the years; however, only a handful of virus- or disease-specific compounds (mostly small molecule drugs) have been approved to be used in humans, for example, antiretrovirals (HIV), direct antivirals (hepatitis C virus, HCV), or agents against influenza or herpes virus infections (77). Similarly, although numerous promising peptides have been evaluated as therapeutic agents for multiple diseases, including several FDA-approved peptide drugs against cancer, type 2 diabetes mellitus, immune dysfunction, or hypertension (78), a very limited number of peptide-based antiviral treatments have been approved to date, for example, the HIV fusion inhibitor enfuvirtide (T-20, Fuzeon) (79). In this study, we evaluated and characterized the ability of D-3006, a synthetic host defense peptide derivative of the L-enantiomer form of IDR-1018 (44, 47, 80) to inhibit SARS-CoV-2 replication *in vitro*, showcasing its potential broad antiviral activity against multiple enveloped viruses.

HDPs have been shown to inhibit the replication of bacteria (planktonic cells and biofilm), fungi, and viruses by different mechanisms, with some disrupting membrane bilayer integrity that could lead to cell death (41). Therefore, as with any potential antiviral candidate, we first evaluated the effect of the four synthetic peptides (D-3006, D-3007, DJK-5, or IDR-1018) in cell viability and proliferation. Similar to previous studies using other cell culture models, including Vero cells (27, 45, 47), none of the HDPs were toxic even at a relatively high concentration of 100 µg/mL in human-derived Caco-2, Huh-7, or Calu-3 cells, a first step to ensure they are safe for potential therapeutic use. In fact, their CC_50_ values ranged from 430 to 708 µg/mL in VeroE6/TMPRSS2 cells, the cell line used to determine their antiviral activity. All four HDPs have been shown to inhibit bacteria replication and have antibiofilm activity (41, 44, 47, 79, and 80, unpublished results); however, only IDR-1018 has been tested as an antiviral, shown to block HSV-2 replication in cell culture and in mice (27). Here, we showed that IDR-1018, as well as the three D-enantiomeric peptides (DJK-5, D-3006, and D-3007), inhibited SARS-CoV-2 replication *in vitro*, with D-3006 being the most potent (EC_50_ values ranging from 1.57 µg/mL to 5.37 µg/mL, depending on the system used). The anti-SARS-CoV-2 potency of D-3006 was similar to that described for other antimicrobial peptides such as LL-37, HNP1, HD5, hβD-2, RC-101, brilacidin, DP7, and P9R (EC_50_ values from 0.6 µg/mL to 14 µg/mL) (24, 29–33, 36–38), resulting in an excellent selectivity index comparable to that of the FDA-approved remdesivir. Moreover, we showed a synergistic effect when combining D-3006 and remdesivir, potently blocking the replication of authentic SARS-CoV-2 *in vitro*, similar to the combination of remdesivir with brilacidin, a small-molecule mimetic of HDPs (37).

The synergistic effect of D-3006 and remdesivir suggested that the synthetic peptide may not be inhibiting the activity of the SARS-CoV-2 RNA-dependent RNA polymerase, the target of remdesivir (71). Thus, to help identify the mechanism of action of D-3006, we first performed a time-of-drug addition assay, showing that the HDP reduced virus replication when added before or simultaneously to infection with authentic SARS-CoV-2, which suggested that the synthetic peptide was interfering with viral entry. The ability of D-3006 to block viral entry was verified using a SARS-CoV-2 spike pseudotyped virus system, showing that the synthetic peptide neutralized a lentivirus pseudotyped with the spike from the ancestral Wuhan SARS-CoV-2 strain. Interestingly, D-3006 interfered with the binding of the SARS-CoV-2 RBD to its human ACE2 receptor but with less affinity (IC_50_ of 131.3 µg/mL or 84 µM) than soluble hACE2 (4.2 µM) and other peptides shown to target this virus replication step, e.g., LL-37 (25.5 nM) (29), HD5 (76.2 nM) (32), HNP1 (160 nM) (81), DP7 (227 nM) (38), hβD-2 (2 µM) (33), and micasin (a fungal defensin, 5 µM) (34). This suggests that although D-3006 seems to hinder the binding of SARS-CoV-2 to its cell receptor, it may also potently block viral entry by a different mechanism.

Host defense synthetic peptides can inhibit virus replication by (i) direct interaction, that is, targeting the viral membrane or virucidal effect; (ii) blocking viral entry, including binding, attachment, and fusion; (iii) interfering with post-entry and intracellular steps in the viral replication cycle; and (iv) modulation of the innate and/or adaptive immune response to aid clearing the viral infection from the host (41, 48, 82, 83). The most common antiviral mechanism of action of HDPs is their ability to destabilize the viral membrane, damaging the integrity of the virion and therefore inhibiting virus infectivity (48). For example, melittin damages the viral membrane, from pore formation to complete membrane lysis (68). LL-37 and other cathelicidins have been shown to bind and damage the virions of Influenza virus, Respiratory syncytial virus, Zika virus, vaccinia virus, human herpesvirus 8 (48). HNP and human β-defensins also disrupt the HIV-1 membrane (19, 84), whereas brilacidin has been shown to disrupt viral integrity and entry of SARS-CoV-2 (37).

The mechanism of action of two of the HDPs described here has been evaluated in bacterial infections: IDR-1018 modulates human neutrophil functions (44, 47, 85) and DJK-5 inhibited biofilm formation by triggering the degradation of intracellular nucleotide ppGpp (86). In addition, IDR-1018 was shown to reduce herpes simplex virus type 2 (HSV-2) infectivity in mice by interfering with viral attachment and entry (27). This is the first study aimed to evaluate and characterize the mode of action of the synthetic host defense peptide D-3006 against SARS-CoV-2. We showed that, unlike shACE2, pre-incubation with D-3006 inhibited entry of viruses pseudotyped with SARS-CoV-2 spike glycoprotein or VSV-g envelope protein in a dose-dependent manner, suggesting that viral entry inhibition may not be specific to blocking the interaction of the SARS-CoV-2 spike with its ACE2 cell receptor. Electron microscopy analysis showed that melittin reduced the number of intact virions, as previously described for SARS-CoV-2 and other viruses (16, 68); however, D-3006 did not significantly affect the integrity of the SARS-CoV-2 membrane. In addition to viral membrane disruption, another common mechanism of action against many enveloped viruses is the capacity of antiviral peptides to bind to virions and mediate aggregation of viral particles, such as influenza virus (HNP1, HD5, P9, and retrocyclins) (26, 48, 82, 87). It is possible that D-3006 binds to the SARS-CoV-2 membrane, interfering with viral attachment and further entry into the cell. This could also explain a potential destabilization of the SARS-CoV-2 spike, which could have an indirect and non-specific effect on the RBD-ACE2 interaction, as observed in the binding assay. Interestingly, we observed a similar effect when IAV was exposed to the synthetic peptide, that is, D-3006 inhibited IAV replication with no detectable effect on the integrity of the membrane of influenza A virus particles, suggesting non-specific binding of the synthetic host defense peptide to viral membranes.

The antiviral activity of D-3006 was not restricted to the authentic SARS-CoV-2 ancestral (Wuhan) strain, but the synthetic peptide was able to inhibit the replication of other authentic SARS-CoV-2 strains (e.g., Delta, Mu, Omicron BA.5), lentiviruses pseudotyped with spike proteins from multiple SARS-CoV-2 variants of concern (Alpha to Omicron), SARS-CoV and even MERS-CoV, as well as replication competent influenza A virus. Other peptides have shown a broad antiviral spectrum, inhibiting the replication of multiple enveloped viruses using different mechanisms (20, 22, 82, 87). For example, α- and β-defensins, as well as retrocyclins, inhibit influenza virus, HIV-1, and SARS-CoV-2 (19, 23, 31, 33, 48, 87, 88), the defensin-like P9 has potent antiviral activity against influenza viruses (H1N1, H7N9) and coronaviruses (SARS-CoV-2, MERS-CoV, and SARS-CoV-2) (24), a frog-defensin-derived peptide broadly inhibits influenza virus and SARS-CoV-2 (35), and brilacidin showed broad-spectrum antiviral activity against SARS-CoV-2 and other human coronaviruses (HCoV-229E, HCoV-OC43, and HCoV-NL63) (89). Here, we showed that similar to other synthetic host defense peptides such as IDR-1018, DJK-5, and DJK-6, which have shown a wide range of antibacterial and antibiofilm activity against both gram-positive and gram-negative bacteria (41, 44, 47, 90), D-3006 was able to inhibit replication of at least two enveloped viruses: SARS-CoV-2 and influenza A virus.

Although the most potent antiviral drugs in the market target viral enzymes, such as polymerases or protease inhibitors (77, 91), the best prophylactic drugs are those blocking viral entry, which are usually effective in preventing or even treating the early phase of viral diseases. This was evident during the COVID-19 pandemic, where monoclonal antibodies targeting the SARS-CoV-2 spike protein were instrumental in the early to mid-stages of the pandemic before vaccines were widely available (9). Due to their efficacy, safety, and potency, antiviral peptides have regained increasing interest as both therapeutic and prophylactic agents (20, 82), with at least one peptide-based antiviral reaching phase 2 clinical trial as a potential treatment for SARS-CoV-2 infection (brilacidin, https://clinicaltrials.gov/study/NCT04784897). Here, we describe the ability of a synthetic host defense peptide, D-3006, to safely and potently inhibit SARS-CoV-2 replication *in vitro*, by interfering with viral attachment and entry. The mechanism of action of D-3006 seems to be associated with non-specific binding to the viral membrane, perhaps facilitated by the presence in the cellular membrane of negatively charged phospholipids, most likely causing virus aggregation. The potential broad-spectrum antiviral activity of the long-lasting D-enantiomer D-3006, particularly against enveloped viruses, as well as the possibility to use it in combination with other antiviral drugs to enhance their efficacy at lower concentrations, warrant further studies to evaluate the effectiveness and safety of this synthetic host defense peptide in prophylactic and/or therapeutic antiviral treatments.

## Data Availability

Raw data can be requested by contacting the corresponding author.

## SUPPLEMENTAL MATERIAL

Supplemental figures aac.01700-24-s0001.pdf Fig. S1 to S7.

ASM does not own the copyrights to Supplemental Material that may be linked to, or accessed through, an article. The authors have granted ASM a non-exclusive, world-wide license to publish the Supplemental Material files. Please contact the corresponding author directly for reuse.

## References

[1] Zhu N, Zhang D, Wang W, Li X, Yang B, Song J, Zhao X, Huang B, Shi W, Lu R, Niu P, Zhan F, Ma X, Wang D, Xu W, Wu G, Gao GF, Tan W, China Novel Coronavirus Investigating and Research Team. 2020. A novel coronavirus from patients with pneumonia in China, 2019. N Engl J Med 382:727–733. doi:10.1056/NEJMoa2001017 31978945 PMC7092803

[2] Baker MG, Wilson N, Anglemyer A. 2020. Successful elimination of covid-19 transmission in New Zealand. N Engl J Med 383:e56. doi:10.1056/NEJMc2025203 32767891 PMC7449141

[3] Al-Jighefee HT, Najjar H, Ahmed MN, Qush A, Awwad S, Kamareddine L. 2021. COVID-19 vaccine platforms: challenges and safety contemplations. Vaccines (Basel) 9:1196. doi:10.3390/vaccines9101196 34696306 PMC8537163

[4] Worobey M, Pekar J, Larsen BB, Nelson MI, Hill V, Joy JB, Rambaut A, Suchard MA, Wertheim JO, Lemey P. 2020. The emergence of SARS-CoV-2 in Europe and North America. Science 370:564–570. doi:10.1126/science.abc8169 32912998 PMC7810038

[5] Wu N, Joyal-Desmarais K, Ribeiro PAB, Vieira AM, Stojanovic J, Sanuade C, Yip D, Bacon SL. 2023. Long-term effectiveness of COVID-19 vaccines against infections, hospitalisations, and mortality in adults: findings from a rapid living systematic evidence synthesis and meta-analysis up to December, 2022. Lancet Respir Med 11:439–452. doi:10.1016/S2213-2600(23)00015-2 36780914 PMC9917454

[6] Williams BA, Jones CH, Welch V, True JM. 2023. Outlook of pandemic preparedness in a post-COVID-19 world. NPJ Vaccines 8:178. doi:10.1038/s41541-023-00773-0 37985781 PMC10662147

[7] Dong E, Du H, Gardner L. 2020. An interactive web-based dashboard to track COVID-19 in real time. Lancet Infect Dis 20:533–534. doi:10.1016/S1473-3099(20)30120-1 32087114 PMC7159018

[8] Li G, Hilgenfeld R, Whitley R, De Clercq E. 2023. Therapeutic strategies for COVID-19: progress and lessons learned. Nat Rev Drug Discov 22:449–475. doi:10.1038/s41573-023-00672-y 37076602 PMC10113999

[9] Focosi D, Franchini M, Maggi F, Shoham S. 2024. COVID-19 therapeutics. Clin Microbiol Rev 37:e0011923. doi:10.1128/cmr.00119-23 38771027 PMC11237566

[10] Chan JF-W, Yuan S, Chu H, Sridhar S, Yuen K-Y. 2024. COVID-19 drug discovery and treatment options. Nat Rev Microbiol 22:391–407. doi:10.1038/s41579-024-01036-y 38622352

[11] Lehrer RI, Lichtenstein AK, Ganz T. 1993. Defensins: antimicrobial and cytotoxic peptides of mammalian cells. Annu Rev Immunol 11:105–128. doi:10.1146/annurev.iy.11.040193.000541 8476558

[12] Ganz T, Lehrer RI. 1998. Antimicrobial peptides of vertebrates. Curr Opin Immunol 10:41–44. doi:10.1016/S0952-7915(98)80029-0 9523109

[13] Yang D, Chertov O, Oppenheim JJ. 2001. The role of mammalian antimicrobial peptides and proteins in awakening of innate host defenses and adaptive immunity. CMLS, Cell Mol Life Sci 58:978–989. doi:10.1007/PL00000914 11497243 PMC11337368

[14] Bevins CL. 2003. Antimicrobial peptides as effector molecules of mammalian host defense, p 106–148. In Herwald H (ed), Host respponse mechanisms in infectious diseases. Basel, Karger. 10.1159/000068134 12530324

[15] Diamond G, Beckloff N, Weinberg A, Kisich KO. 2009. The roles of antimicrobial peptides in innate host defense. Curr Pharm Des 15:2377–2392. doi:10.2174/138161209788682325 19601838 PMC2750833

[16] Yasin B, Pang M, Turner JS, Cho Y, Dinh NN, Waring AJ, Lehrer RI, Wagar EA. 2000. Evaluation of the inactivation of infectious herpes simplex virus by host-defense peptides. Eur J Clin Microbiol Infect Dis 19:187–194. doi:10.1007/s100960050457 10795591

[17] Münk C, Wei G, Yang OO, Waring AJ, Wang W, Hong T, Lehrer RI, Landau NR, Cole AM. 2003. The θ-defensin, retrocyclin, inhibits HIV-1 entry. AIDS Res Hum Retroviruses 19:875–881. doi:10.1089/088922203322493049 14585219

[18] Mackewicz CE, Yuan J, Tran P, Diaz L, Mack E, Selsted ME, Levy JA. 2003. Alpha-Defensins can have anti-HIV activity but are not CD8 cell anti-HIV factors. AIDS 17:F23–32. doi:10.1097/00002030-200309260-00001 14502030

[19] Quiñones-Mateu ME, Lederman MM, Feng Z, Chakraborty B, Weber J, Rangel HR, Marotta ML, Mirza M, Jiang B, Kiser P, Medvik K, Sieg SF, Weinberg A. 2003. Human epithelial beta-defensins 2 and 3 inhibit HIV-1 replication. AIDS 17:F39–48. doi:10.1097/00002030-200311070-00001 14571200

[20] Vilas Boas LCP, Campos ML, Berlanda RLA, de Carvalho Neves N, Franco OL. 2019. Antiviral peptides as promising therapeutic drugs. Cell Mol Life Sci 76:3525–3542. doi:10.1007/s00018-019-03138-w 31101936 PMC7079787

[21] Daher KA, Selsted ME, Lehrer RI. 1986. Direct inactivation of viruses by human granulocyte defensins. J Virol 60:1068–1074. doi:10.1128/jvi.60.3.1068-1074.1986 3023659 PMC253347

[22] Gwyer Findlay E, Currie SM, Davidson DJ. 2013. Cationic host defence peptides: potential as antiviral therapeutics. BioDrugs 27:479–493. doi:10.1007/s40259-013-0039-0 23649937 PMC3775153

[23] Park MS, Kim JI, Lee I, Park S, Bae JY, Park MS. 2018. Towards the application of human defensins as antivirals. Biomolecules Therapeutics 26:242–254. doi:10.4062/biomolther.2017.172 29310427 PMC5933891

[24] Zhao H, To KKW, Sze K-H, Yung TT-M, Bian M, Lam H, Yeung ML, Li C, Chu H, Yuen K-Y. 2020. A broad-spectrum virus- and host-targeting peptide against respiratory viruses including influenza virus and SARS-CoV-2. Nat Commun 11:4252. doi:10.1038/s41467-020-17986-9 32843628 PMC7447754

[25] Li Q, Zhao Z, Zhou D, Chen Y, Hong W, Cao L, Yang J, Zhang Y, Shi W, Cao Z, Wu Y, Yan H, Li W. 2011. Virucidal activity of a scorpion venom peptide variant mucroporin-M1 against measles, SARS-CoV and influenza H5N1 viruses. Peptides 32:1518–1525. doi:10.1016/j.peptides.2011.05.015 21620914 PMC7115635

[26] Hoffmann AR, Guha S, Wu E, Ghimire J, Wang Y, He J, Garry RF, Wimley WC. 2020. Broad-spectrum antiviral entry inhibition by interfacially active peptides. J Virol 94:e01682-20. doi:10.1128/JVI.01682-20 32907984 PMC7654261

[27] Shestakov A, Jenssen H, Hancock REW, Nordström I, Eriksson K. 2013. Synthetic analogues of bovine bactenecin dodecapeptide reduce herpes simplex virus type 2 infectivity in mice. Antiviral Res 100:455–459. doi:10.1016/j.antiviral.2013.08.019 24012999

[28] Tonk M, Růžek D, Vilcinskas A. 2021. Compelling evidence for the activity of antiviral peptides against SARS-CoV-2. Viruses 13:912. doi:10.3390/v13050912 34069206 PMC8156787

[29] Wang C, Wang S, Li D, Chen P, Han S, Zhao G, Chen Y, Zhao J, Xiong J, Qiu J, Wei DQ, Zhao J, Wang J. 2021. Human cathelicidin inhibits SARS-CoV-2 infection: killing two birds with one stone. ACS Infect Dis 7:1545–1554. doi:10.1021/acsinfecdis.1c00096 33849267

[30] Li D, Chen P, Shi T, Mehmood A, Qiu J. 2021. HD5 and LL-37 Inhibit SARS-CoV and SARS-CoV-2 binding to human ACE2 by molecular simulation. Interdiscip Sci 13:766–777. doi:10.1007/s12539-021-00462-3 34363600 PMC8346780

[31] Xu C, Wang A, Marin M, Honnen W, Ramasamy S, Porter E, Subbian S, Pinter A, Melikyan GB, Lu W, Chang TL. 2021. Human defensins inhibit SARS-CoV-2 infection by blocking viral entry. Viruses 13:1246. doi:10.3390/v13071246 34206990 PMC8310277

[32] Wang C, Wang S, Li D, Wei DQ, Zhao J, Wang J. 2020. Human Intestinal Defensin 5 Inhibits SARS-CoV-2 Invasion by Cloaking ACE2. Gastroenterology 159:1145–1147. doi:10.1053/j.gastro.2020.05.015 32437749 PMC7211585

[33] Zhang L, Ghosh SK, Basavarajappa SC, Chen Y, Shrestha P, Penfield J, Brewer A, Ramakrishnan P, Buck M, Weinberg A. 2022. HBD-2 binds SARS-CoV-2 RBD and blocks viral entry: Strategy to combat COVID-19. iScience 25:103856. doi:10.1016/j.isci.2022.103856 35128350 PMC8808565

[34] Gao B, Zhu S. 2021. A fungal defensin targets the SARS-CoV-2 spike receptor-binding domain. J Fungi (Basel) 7:553. doi:10.3390/jof7070553 34356932 PMC8304516

[35] Zhao H, Meng X, Peng Z, Lam H, Zhang C, Zhou X, Chan JF-W, Kao RYT, To KK-W, Yuen K-Y. 2022. Fusion-inhibition peptide broadly inhibits influenza virus and SARS-CoV-2, including Delta and Omicron variants. Emerging Microbes & Infections 11:926–937. doi:10.1080/22221751.2022.2051753 35259078 PMC8973381

[36] Kudryashova E, Zani A, Vilmen G, Sharma A, Lu W, Yount JS, Kudryashov DS. 2022. Inhibition of SARS-CoV-2 infection by human defensin HNP1 and retrocyclin RC-101. J Mol Biol 434:167225. doi:10.1016/j.jmb.2021.167225 34487793 PMC8413479

[37] Bakovic A, Risner K, Bhalla N, Alem F, Chang TL, Weston WK, Harness JA, Narayanan A. 2021. Brilacidin demonstrates inhibition of SARS-CoV-2 in cell culture. Viruses 13:271. doi:10.3390/v13020271 33572467 PMC7916214

[38] Zhang R, Jiang X, Qiao J, Wang Z, Tong A, Yang J, Yang S, Yang L. 2021. Antimicrobial peptide DP7 with potential activity against SARS coronavirus infections. Signal Transduct Target Ther 6:140. doi:10.1038/s41392-021-00551-1 33795636 PMC8012516

[39] de Vries RD, Schmitz KS, Bovier FT, Predella C, Khao J, Noack D, Haagmans BL, Herfst S, Stearns KN, Drew-Bear J, Biswas S, Rockx B, McGill G, Dorrello NV, Gellman SH, Alabi CA, de Swart RL, Moscona A, Porotto M. 2021. Intranasal fusion inhibitory lipopeptide prevents direct-contact SARS-CoV-2 transmission in ferrets. Science 371:1379–1382. doi:10.1126/science.abf4896 33597220 PMC8011693

[40] Wang X, Xia S, Wang Q, Xu W, Li W, Lu L, Jiang S. 2020. Broad-spectrum coronavirus fusion inhibitors to combat COVID-19 and other emerging coronavirus disefases. IJMS 21:3843. doi:10.3390/ijms21113843 32481690 PMC7311999

[41] Hancock REW, Alford MA, Haney EF. 2021. Antibiofilm activity of host defence peptides: complexity provides opportunities. Nat Rev Microbiol 19:786–797. doi:10.1038/s41579-021-00585-w 34183822

[42] Chongsiriwatana NP, Lin JS, Kapoor R, Wetzler M, Rea JAC, Didwania MK, Contag CH, Barron AE. 2017. Intracellular biomass flocculation as a key mechanism of rapid bacterial killing by cationic, amphipathic antimicrobial peptides and peptoids. Sci Rep 7:16718. doi:10.1038/s41598-017-16180-0 29196622 PMC5711933

[43] de la Fuente-Núñez C, Reffuveille F, Haney EF, Straus SK, Hancock REW. 2014. Broad-spectrum anti-biofilm peptide that targets a cellular stress response. PLoS Pathog 10:e1004152. doi:10.1371/journal.ppat.1004152 24852171 PMC4031209

[44] Mansour SC, de la Fuente-Núñez C, Hancock REW. 2015. Peptide IDR-1018: modulating the immune system and targeting bacterial biofilms to treat antibiotic-resistant bacterial infections. J Pept Sci 21:323–329. doi:10.1002/psc.2708 25358509

[45] Wu BC, Haney EF, Akhoundsadegh N, Pletzer D, Trimble MJ, Adriaans AE, Nibbering PH, Hancock REW. 2021. Human organoid biofilm model for assessing antibiofilm activity of novel agents. NPJ Biofilms Microbiomes 7:8. doi:10.1038/s41522-020-00182-4 33495449 PMC7835231

[46] Vaara M. 1992. Agents that increase the permeability of the outer membrane. Microbiol Rev 56:395–411. doi:10.1128/mr.56.3.395-411.1992 1406489 PMC372877

[47] Niyonsaba F, Madera L, Afacan N, Okumura K, Ogawa H, Hancock REW. 2013. The innate defense regulator peptides IDR-HH2, IDR-1002, and IDR-1018 modulate human neutrophil functions. J Leukoc Biol 94:159–170. doi:10.1189/jlb.1012497 23616580

[48] Mookherjee N, Anderson MA, Haagsman HP, Davidson DJ. 2020. Antimicrobial host defence peptides: functions and clinical potential. Nat Rev Drug Discov 19:311–332. doi:10.1038/s41573-019-0058-8 32107480

[49] de la Fuente-Núñez C, Cardoso MH, de Souza Cândido E, Franco OL, Hancock REW. 2016. Synthetic antibiofilm peptides. Biochim Biophys Acta 1858:1061–1069. doi:10.1016/j.bbamem.2015.12.015 26724202 PMC4809770

[50] Wang D, Shen Y, Hancock REW, Ma J, Haapasalo M. 2018. Antimicrobial effect of peptide DJK-5 used alone or mixed with EDTA on mono- and multispecies biofilms in dentin canals. J Endod 44:1709–1713. doi:10.1016/j.joen.2018.07.018 30243660

[51] Crabbé A, Liu Y, Matthijs N, Rigole P, De La Fuente-Nùñez C, Davis R, Ledesma MA, Sarker S, Van Houdt R, Hancock REW, Coenye T, Nickerson CA. 2017. Antimicrobial efficacy against Pseudomonas aeruginosa biofilm formation in a three-dimensional lung epithelial model and the influence of fetal bovine serum. Sci Rep 7:43321. doi:10.1038/srep43321 28256611 PMC5335707

[52] Mansour SC, Pletzer D, de la Fuente-Núñez C, Kim P, Cheung GYC, Joo H-S, Otto M, Hancock REW. 2016. Bacterial abscess formation is controlled by the stringent stress response and can be targeted therapeutically. EBioMedicine 12:219–226. doi:10.1016/j.ebiom.2016.09.015 27658736 PMC5078632

[53] Pletzer D, Mansour SC, Hancock REW. 2018. Synergy between conventional antibiotics and anti-biofilm peptides in a murine, sub-cutaneous abscess model caused by recalcitrant ESKAPE pathogens. PLoS Pathog 14:e1007084. doi:10.1371/journal.ppat.1007084 29928049 PMC6013096

[54] Yamada S, Fukushi S, Kinoshita H, Ohnishi M, Suzuki T, Fujimoto T, Saijo M, Maeda K. 2021. Assessment of SARS-CoV-2 infectivity of upper respiratory specimens from COVID-19 patients by virus isolation using VeroE6/TMPRSS2 cells. BMJ Open Resp Res 8:e000830. doi:10.1136/bmjresp-2020-000830 PMC7907832 33627333

[55] Li Y, Larrimer A, Curtiss T, Kim J, Jones A, Baird-Tomlinson H, Pekosz A, Olivo PD. 2009. Influenza virus assays based on virus-inducible reporter cell lines. Influenza Other Respir Viruses 3:241–251. doi:10.1111/j.1750-2659.2009.00095.x 21462401 PMC4940803

[56] Harfoot R, Lawley B, Hernández LC, Kuang J, Grant J, Treece JM, LeQueux S, Day R, Jack S, Stanton J-AL, Bostina M, Ussher JE, Quiñones-Mateu ME. 2022. Characterization of the first SARS-CoV-2 isolates from aotearoa New Zealand as part of a rapid response to the COVID-19 pandemic. Viruses 14:366. doi:10.3390/v14020366 35215963 PMC8877023

[57] Reed LJ, Muench H. 1938. A simple method of estimating fifty per cent endpoints12. AmJHyg 27:493–497. doi:10.1093/oxfordjournals.aje.a118408

[58] Ranaivoson FM, Turk LS, Ozgul S, Kakehi S, von Daake S, Lopez N, Trobiani L, De Jaco A, Denissova N, Demeler B, Özkan E, Montelione GT, Comoletti D. 2019. A proteomic screen of neuronal cell-surface molecules reveals IgLONs as structurally conserved interaction modules at the synapse. Structure 27:893–906. doi:10.1016/j.str.2019.03.004 30956130 PMC6609445

[59] Rubio-Marrero EN, Vincelli G, Jeffries CM, Shaikh TR, Pakos IS, Ranaivoson FM, von Daake S, Demeler B, De Jaco A, Perkins G, Ellisman MH, Trewhella J, Comoletti D. 2016. Structural characterization of the extracellular domain of CASPR2 and insights into its association with the novel ligand contactin1. J Biol Chem 291:5788–5802. doi:10.1074/jbc.M115.705681 26721881 PMC4786715

[60] Comoletti D, Miller MT, Jeffries CM, Wilson J, Demeler B, Taylor P, Trewhella J, Nakagawa T. 2010. The macromolecular architecture of extracellular domain of alphaNRXN1: domain organization, flexibility, and insights into trans-synaptic disposition. Structure 18:1044–1053. doi:10.1016/j.str.2010.06.005 20696403 PMC2948785

[61] Montgomerie I, Bird TW, Palmer OR, Mason NC, Pankhurst TE, Lawley B, Hernández LC, Harfoot R, Authier-Hall A, Anderson DE, et al.. 2023. Incorporation of SARS-CoV-2 spike NTD to RBD protein vaccine improves immunity against viral variants. iScience 26:106256. doi:10.1016/j.isci.2023.106256 36845030 PMC9940465

[62] Lawley B, Grant J, Harfoot R, Treece JM, Day R, Hernández LC, Stanton J-AL, Ussher JE, Quiñones-Mateu ME. 2021. Rapid Response to SARS-CoV-2 in Aotearoa New Zealand: implementation of a diagnostic test and characterization of the first COVID-19 cases in the South Island. Viruses 13:2222. doi:10.3390/v13112222 34835031 PMC8623489

[63] Crawford KHD, Eguia R, Dingens AS, Loes AN, Malone KD, Wolf CR, Chu HY, Tortorici MA, Veesler D, Murphy M, Pettie D, King NP, Balazs AB, Bloom JD. 2020. Protocol and reagents for pseudotyping lentiviral particles with SARS-CoV-2 spike protein for neutralization assays. Viruses 12:513. doi:10.3390/v12050513 32384820 PMC7291041

[64] Shu Y, McCauley J. 2017. GISAID: global initiative on sharing all influenza data - from vision to reality. Euro Surveill 22:30494. doi:10.2807/1560-7917.ES.2017.22.13.30494 28382917 PMC5388101

[65] Larkin MA, Blackshields G, Brown NP, Chenna R, McGettigan PA, McWilliam H, Valentin F, Wallace IM, Wilm A, Lopez R, Thompson JD, Gibson TJ, Higgins DG. 2007. Clustal W and Clustal X version 2.0. Bioinformatics 23:2947–2948. doi:10.1093/bioinformatics/btm404 17846036

[66] Tamura K, Peterson D, Peterson N, Stecher G, Nei M, Kumar S. 2011. MEGA5: molecular evolutionary genetics analysis using maximum likelihood, evolutionary distance, and maximum parsimony methods. Mol Biol Evol 28:2731–2739. doi:10.1093/molbev/msr121 21546353 PMC3203626

[67] Ianevski A, Giri AK, Aittokallio T. 2020. SynergyFinder 2.0: visual analytics of multi-drug combination synergies. Nucleic Acids Res 48:W488–W493. doi:10.1093/nar/gkaa216 32246720 PMC7319457

[68] Enayathullah MG, Parekh Y, Banu S, Ram S, Nagaraj R, Kumar BK, Idris MM. 2022. Gramicidin S and melittin: potential anti-viral therapeutic peptides to treat SARS-CoV-2 infection. Sci Rep 12:3446. doi:10.1038/s41598-022-07341-x 35236909 PMC8891299

[69] Trubiani O, Ciancarelli M, Rapino M, Di Primio R. 1996. Dimethyl sulfoxide induces programmed cell death and reversible G1 arrest in the cell cycle of human lymphoid pre-T cell line. Immunol Lett 50:51–57. doi:10.1016/0165-2478(96)02518-7 8793559

[70] Matsuyama S, Nao N, Shirato K, Kawase M, Saito S, Takayama I, Nagata N, Sekizuka T, Katoh H, Kato F, Sakata M, Tahara M, Kutsuna S, Ohmagari N, Kuroda M, Suzuki T, Kageyama T, Takeda M. 2020. Enhanced isolation of SARS-CoV-2 by TMPRSS2-expressing cells. Proc Natl Acad Sci USA 117:7001–7003. doi:10.1073/pnas.2002589117 32165541 PMC7132130

[71] Malin JJ, Suárez I, Priesner V, Fätkenheuer G, Rybniker J. 2020. Remdesivir against COVID-19 and other viral diseases. Clin Microbiol Rev 34:e00162-20. doi:10.1128/CMR.00162-20 33055231 PMC7566896

[72] Jackson CB, Farzan M, Chen B, Choe H. 2022. Mechanisms of SARS-CoV-2 entry into cells. Nat Rev Mol Cell Biol 23:3–20. doi:10.1038/s41580-021-00418-x 34611326 PMC8491763

[73] Harvey WT, Carabelli AM, Jackson B, Gupta RK, Thomson EC, Harrison EM, Ludden C, Reeve R, Rambaut A, COVID-19 Genomics UK (COG-UK) Consortium, Peacock SJ, Robertson DL. 2021. SARS-CoV-2 variants, spike mutations and immune escape. Nat Rev Microbiol 19:409–424. doi:10.1038/s41579-021-00573-0 34075212 PMC8167834

[74] Li J, Lai S, Gao GF, Shi W. 2021. The emergence, genomic diversity and global spread of SARS-CoV-2. Nature 600:408–418. doi:10.1038/s41586-021-04188-6 34880490

[75] Choi JA, Wu K, Kim GN, Saeedian N, Seon SH, Park G, Jung DI, Jeong HW, Kim NH, Seo SH, Lee S, Song M, Kang CY. 2021. Induction of protective immune responses against a lethal Zika virus challenge post-vaccination with a dual serotype of recombinant vesicular stomatitis virus carrying the genetically modified Zika virus E protein gene. J Gen Virol 102. doi:10.1099/jgv.0.001588 33913804

[76] Li W, Moore MJ, Vasilieva N, Sui J, Wong SK, Berne MA, Somasundaran M, Sullivan JL, Luzuriaga K, Greenough TC, Choe H, Farzan M. 2003. Angiotensin-converting enzyme 2 is a functional receptor for the SARS coronavirus. Nature 426:450–454. doi:10.1038/nature02145 14647384 PMC7095016

[77] De Clercq E, Li G. 2016. Approved antiviral drugs over the past 50 years. Clin Microbiol Rev 29:695–747. doi:10.1128/CMR.00102-15 27281742 PMC4978613

[78] Wang L, Wang N, Zhang W, Cheng X, Yan Z, Shao G, Wang X, Wang R, Fu C. 2022. Therapeutic peptides: current applications and future directions. Sig Transduct Target Ther 7:48. doi:10.1038/s41392-022-00904-4 PMC8844085 35165272

[79] Matthews T, Salgo M, Greenberg M, Chung J, DeMasi R, Bolognesi D. 2004. Enfuvirtide: the first therapy to inhibit the entry of HIV-1 into host CD4 lymphocytes. Nat Rev Drug Discov 3:215–225. doi:10.1038/nrd1331 15031735

[80] Haney EF, Brito-Sánchez Y, Trimble MJ, Mansour SC, Cherkasov A, Hancock REW. 2018. Computer-aided discovery of peptides that specifically attack bacterial biofilms. Sci Rep 8:1871. doi:10.1038/s41598-018-19669-4 29382854 PMC5789975

[81] Pramanik A, Sharma PC, Patibandla S, Gao Y, Ruppa-Kasani V, Goli J, Kumar A, Chatterjee A, Sinha SS, Bates JT, Bierdeman MA, Tandon R, Ray PC. 2022. Blocking SARS-CoV-2 delta variant (B.1.617.2) spike protein receptor-binding domain binding with the ACE2 receptor of the host cell and inhibiting virus infections using human host defense peptide-conjugated graphene quantum dots. ACS Omega 7:8150–8157. doi:10.1021/acsomega.2c00113 35252734 PMC8886715

[82] Urmi UL, Vijay AK, Kuppusamy R, Islam S, Willcox MDP. 2023. A review of the antiviral activity of cationic antimicrobial peptides. Peptides 166:171024. doi:10.1016/j.peptides.2023.171024 37172781 PMC10170872

[83] Ali W, Elsahn A, Ting DSJ, Dua HS, Mohammed I. 2022. Host defence peptides: a potent alternative to combat antimicrobial resistance in the era of the COVID-19 pandemic. Antibiotics (Basel) 11:475. doi:10.3390/antibiotics11040475 35453226 PMC9032040

[84] Wang W, Owen SM, Rudolph DL, Cole AM, Hong T, Waring AJ, Lal RB, Lehrer RI. 2004. Activity of α- and θ-defensins against primary isolates of HIV-1. JImmunol 173:515–520. doi:10.4049/jimmunol.173.1.515 15210812

[85] Pletzer D, Wolfmeier H, Bains M, Hancock REW. 2017. Synthetic peptides to target stringent response-controlled virulence in a Pseudomonas aeruginosa murine cutaneous infection model. Front Microbiol 8:1867. doi:10.3389/fmicb.2017.01867 29021784 PMC5623667

[86] de la Fuente-Núñez C, Reffuveille F, Mansour SC, Reckseidler-Zenteno SL, Hernández D, Brackman G, Coenye T, Hancock REW. 2015. D-enantiomeric peptides that eradicate wild-type and multidrug-resistant biofilms and protect against lethal Pseudomonas aeruginosa infections. Chem Biol 22:196–205. doi:10.1016/j.chembiol.2015.01.002 25699603 PMC4362967

[87] Solanki SS, Singh P, Kashyap P, Sansi MS, Ali SA. 2021. Promising role of defensins peptides as therapeutics to combat against viral infection. Microb Pathog 155:104930. doi:10.1016/j.micpath.2021.104930 33933603 PMC8084285

[88] Cole AM, Hong T, Boo LM, Nguyen T, Zhao C, Bristol G, Zack JA, Waring AJ, Yang OO, Lehrer RI. 2002. Retrocyclin: a primate peptide that protects cells from infection by T- and M-tropic strains of HIV-1. Proc Natl Acad Sci USA 99:1813–1818. doi:10.1073/pnas.052706399 11854483 PMC122276

[89] Hu Y, Jo H, DeGrado WF, Wang J. 2022. Brilacidin, a COVID-19 drug candidate, demonstrates broad-spectrum antiviral activity against human coronaviruses OC43, 229E, and NL63 through targeting both the virus and the host cell. J Med Virol 94:2188–2200. doi:10.1002/jmv.27616 35080027 PMC8930451

[90] Pletzer D, Hancock REW. 2016. Antibiofilm peptides: potential as broad-spectrum agents. J Bacteriol 198:2572–2578. doi:10.1128/JB.00017-16 27068589 PMC5019066

[91] Clercq E. 2009. The history of antiretrovirals: key discoveries over the past 25 years. Rev Med Virol 19:287–299. doi:10.1002/rmv.624 19714702

