# Supplementary material for: Synthetic host defense peptide inhibits SARS-CoV-2 replication in vitro
Source: Antimicrob Agents Chemother. 2025 Jun 23;69(8):e01700-24. doi: 10.1128/aac.01700-24 (PMC12326988; doi:10.1128/aac.01700-24)
Supplement: Supplemental figures — Fig. S1 to S7. [file aac.01700-24-s0001.pdf]

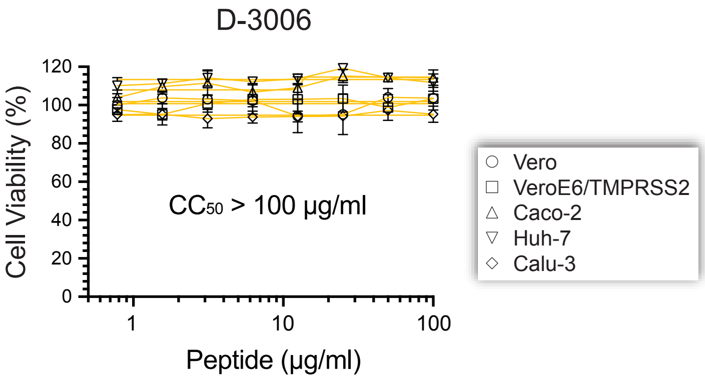

Supplementary Figure 1. Cellular toxicity of D-3006.
Viability of Vero, VeroE6/TMPRSS2, Caco-2, Huh-7, and Calu-3 cells exposed to D-3006. Dose-dependent cytotoxic effect was evaluated for D-3006 in all five cell lines, using serial dilutions of D-3006 starting at 100 µg/ml. No effect in cell viability was observed, in any cell line, at the max concentration of 100 µg/ml.

A. Amino accid sequence of the hACE2 construct

MDSKGSSQKGSRLLLLLVVSNLLLCQGVVS**DYKDDDDK**AAA**QSTIEEQAKTFLDKFNHEAEDLFYQSSLASWNYNTNI**
TEENVQNMNNAGDKWSAFLKEQSTLAQMYPLQEIQNLTVKLQLQALQQNGSSVLSEDKSKRLNTILNTMSTIYSTGKV
CNPDNPQECLLLEPGLNEIMANSLDYNERLWAWESWRSEVGKQLRPLYEEYVVLKNEMARANHYEDYGDYWRGDYEVN
GVDGYDYSRGQLIEDVEHTFEEIKPLYEHLHAYVRAKLMNAYPSYISPIGCLPAHLLGDMWGRFWTNLYSLTVPFGQK
PNIDVTDAMVDQAWDAQRIFKEAEKFFVSVGLPNMTQGFWENSMLTDPGNVQKAVCHPTAWDLGKGDFRILMCTKVTM
DDFLTAHHEMGHIQYDMAYAAQPFLLRNGANEGFHEAVGEIMSLSAATPKHLKSIGLLSPDFQEDNETEINFLLKQAL
TIVGTLPFTYMLEKWRWMVFKGEIPKDQWMKKWWEMKREIVGVVEPVPHDETYCDPASLFHVSNDYSFIRYYTRTLYQ
FQFQEALCQAAKHEGPLHKCDISNSTEAGQKLFNMLRLGKSEPWTLALENVVGAKNMNVRPLLNYFEPLFTWLKDQNK
NSFVGWSTDWSPYADQSIKVRISLKSALGDKAYEWNDNEMYLFRSSVAYAMRQYFLKVKNQMILFGEEDVRVANLKPR
ISFNFFVTAPKNVSDIIPRTEVEKAIRMSRSRINDAFRLNDNSLEFLGIQPTLGPPNQPPVSLEVLFQGPDPDPEEPK
SCDKTHTCPPCPAPELLGGPSVFLFPPKPKDTLMISRTPEVTCVVVDVSHEDPEVKFNWYVDGVEVHNAKTKPREEQY
NSTYRVVSVLTVLHQDWLNGKEYKCKVSNKALPAPIEKTISKAKGQPREPQVYTLPPSRDELTKNQVSLTCLVKGFYP
SDIAVEWESNGQPENNYKTTPPVLDSDGSFFLYSKLTVDKSRWQQGNVFSCSVMHEALHNHYTQKSLSLSPGK*

B. Schematic of plasmid hACE2-Fc

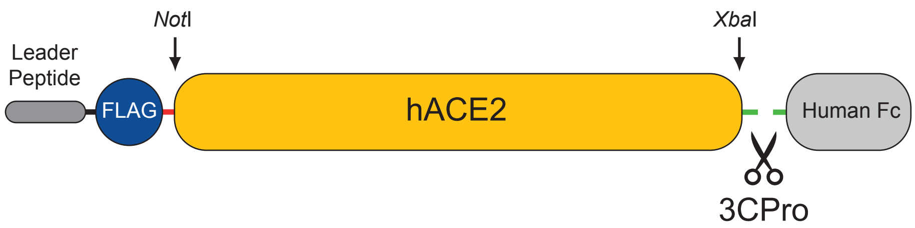

C. Characterization of the secreted dimeric hACE2

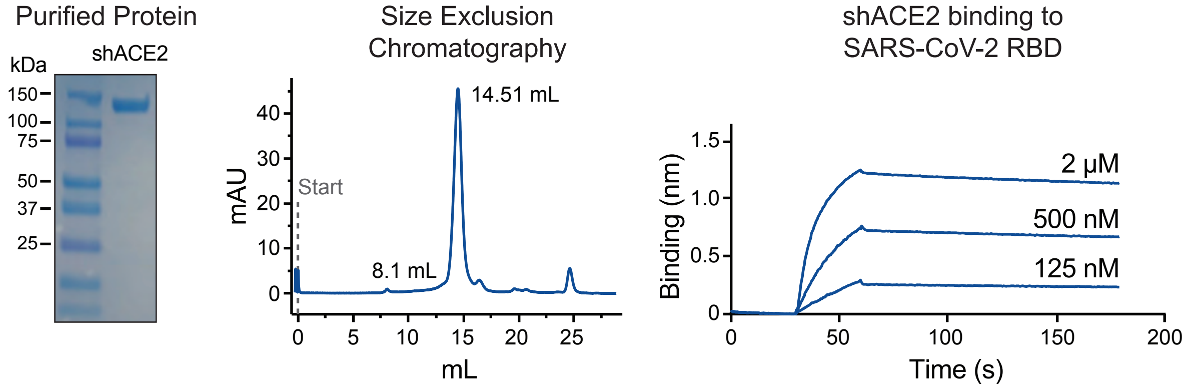

D. Inhibition of hACE2-RBD binding

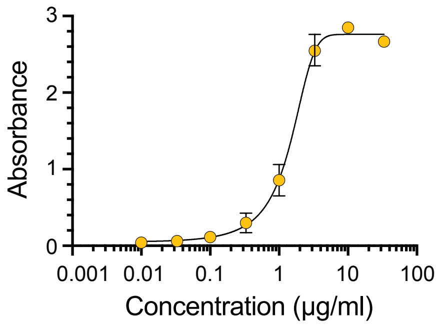

E. Anti-SARS-CoV-2 activity

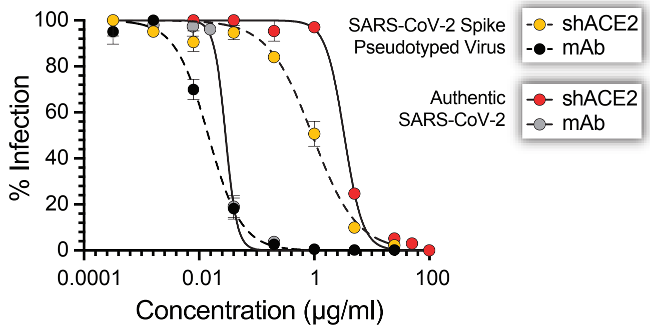

Supplementary Figure 2. Characterization of soluble human ACE2. (A and B) Amino acid sequence and schematic of the human angiotensin converting enzyme 2 (hACE2) cloned in frame with the C-terminal human Fc fragment. The leader peptide (gray), FLAG tag peptide (blue), AlaAlaAla amino acid linker (red), hACE2 extracellular domain (amino acids 18 to 740, black bold text), LeuGluValLeuPheGln ↓ GlyPro amino acid sequence target (green, cleavage site) of the HRV 3C protease, and the C-terminal human Fc fragment (gray), are indicated. *Not*I and *Xba*I restriction enzyme sites used to clone the hACE2 into the modified pCMV6-XL4 vector. (C) Purified soluble hACE2 (shACE2)-Fc protein, prior cleavage with the HRV 3C protease, was verified by Western blot. Size exclusion chromatography after removal of the Fc portion (91) showed that the purified shACE2 protein eluded as a single peak at ~14.5 ml. Minimal aggregation was detected (8.1 ml peak). Binding between the shACE2 and the SARS-CoV-2 (Wuhan) RBD-Fc was assessed by Enzyme-linked immunosorbent assay (ELISA) (91) using three concentrations of purified shACE2 (125 nM, 500 nM, and 2μM) to immobilized SARS-CoV-2 RBD Fc. (D) Competitive ELISA to test the ability of shACE2 to inhibit the binding between SARS-CoV-2 RBD-Fc and hACE2-alkaline phosphatase (AP) fusions. Absorbance was determined at 650 nm. (E) Dose-dependent inhibition of authentic SARS-CoV-2 hCoV-19/New Zealand/NZ1_patient/2020 isolate replication in VeroE6/TMPRSS2 cells and entry of SARS-CoV-2 (Wuhan) spike pseudotyped virus into HEK293T-hACE2 cells by shACE2 or a SARS-CoV-2 spike neutralizing monoclonal antibody (mAb, SinoBiological, Beijing, China).

A. Anti-SARS-CoV-2-Nluc activity of D-3006

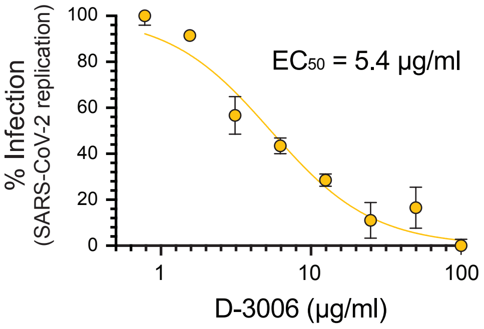

B. D-3006 blocks entry of SARS-CoV-2 expressing mNG fluorescent protein

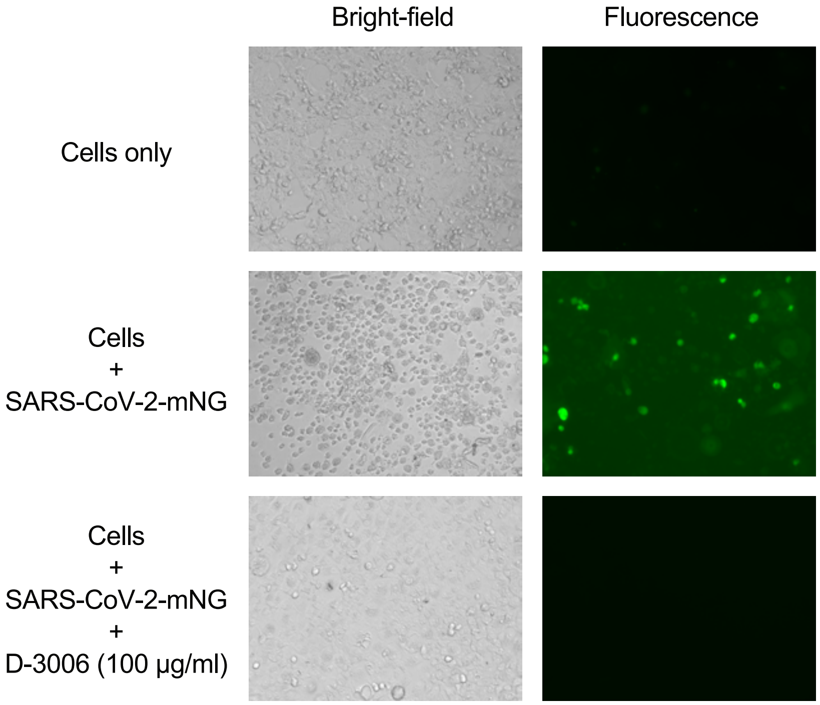

Supplementary Figure 3. Antiviral activity of D-3006.
(A) Susceptibility of the SARS-CoV-2-Nluc expressing the NanoLuc (Nluc, an ATP- independent luciferase producing glow-type luminescence) to D-3006 in VeroE6/TMPRSS2 cells. SARS-CoV-2 replication was quantified 72 hours post-infection by measuring luciferase expression as relative light units (RLU). The EC_{50} value, determined using the non-linear regression model (log inhibitor vs. normalized response-variable slope), is shown. (B) The ability of D-3006 to block SARS-CoV-2 entry was determined by pre-incubating VeroE6/TMPRSS2 cells wth D-3006 for two hours prior infection with SARS-CoV-2-mNG expressing MNeonGreen (a monomeric green or yellow fluorescent protein). As expected, no SARS-CoV-2 infection (no green cells) were observed when the cells were pretreated with the synthetic host defense peptide, supporting the results obtained with the time-of-addition assay (Figure 3).

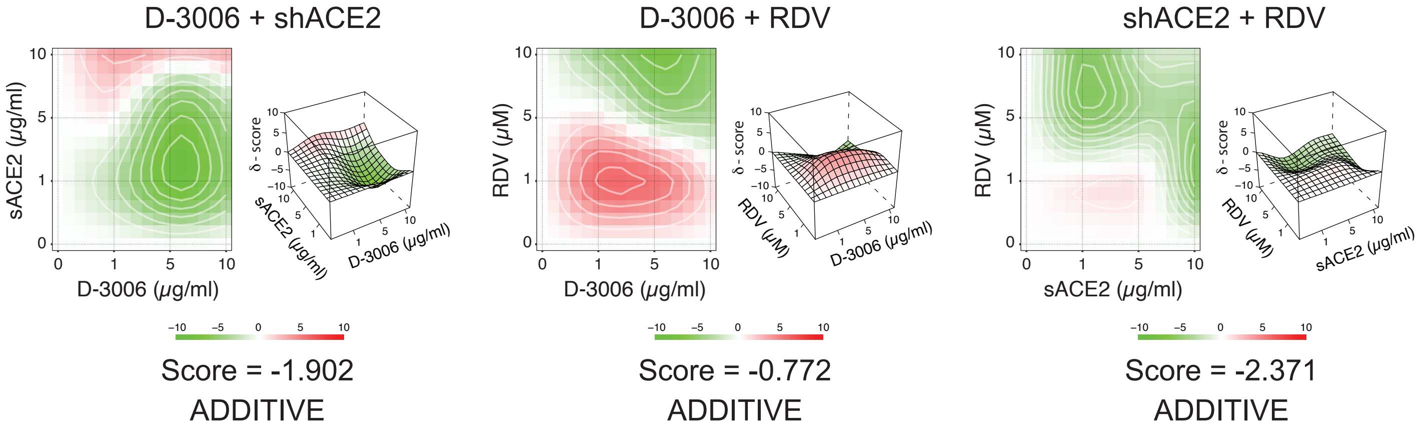

Supplementary Figure 4. Combination treatment of D-3006 with shACE2 or remdesivir. HEK293T-hACE2 cells were infected with SARS-CoV-2 (Wuhan) spike pseudotyped lentivirus in the presence and absence of each agent alone and in combination at the following concentrations: 0, 1, 5, and 10 µg/ml (D-3006 and shACE2) or µM (remdesivir), and the degree of synergistic, additivity, or antagonistic effect quantified using SynergyFinder. Scores for drug combinations were visualized as heatmaps and 3D synergy maps, interpreted as the average excess due to drug interactions. Scores < -10, in the range of -10 to 10, or > 10 suggest that interaction between the two agents is likely to be antagonistic, additive, or synergistic, respectively.

A. Genome Coverage

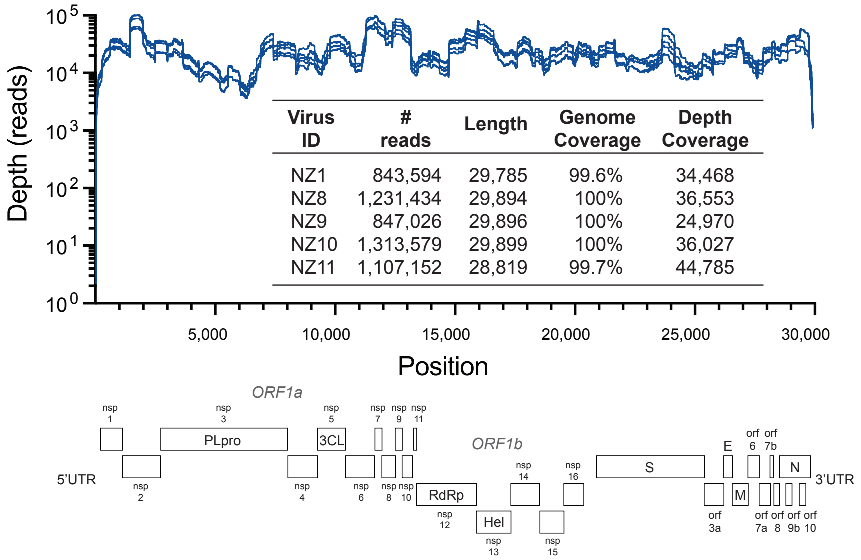

Virus ID	# reads	Length	Genome Coverage	Depth Coverage
NZ1	843,594	29,785	99.6%	34,468
NZ8	1,231,434	29,894	100%	36,553
NZ9	847,026	29,896	100%	24,970
NZ10	1,313,579	29,899	100%	36,027
NZ11	1,107,152	28,819	99.7%	44,785

B. SARS-CoV-2 Phylogeny

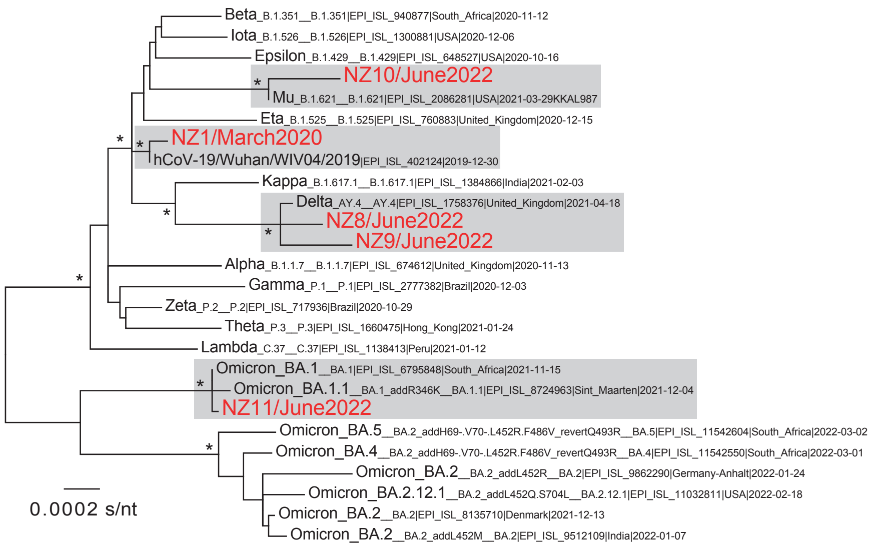

C. Clade/Lineage Classification

Patient ID	Nextrain Clade [a]	Pango Lineage [b]	GISAID Clade [c]	Variant of Concern
NZ1/March2020	19A	B.55	L	Ancestral
NZ8/June2022	21J	AY.23	GK	Delta
NZ9/June2022	21J	AY.76	GK	Delta
NZ10/June2022	21H	B.1.621.1	GH	Mu
NZ11/June2022	21K	BA.1	GRA	Omicron

Supplementary Figure 5. Whole genome sequencing of SARS-CoV-2 isolates from New Zealand in early 2022. (A) Coverage, i.e., number of reads per nucleotide position obtained by deep sequencing the SARS-CoV-2 isolates using the MiSeq platform (Illumina). The position relative to the SARS-CoV-2 isolate Wuhan-Hu-1 NC_045512 is indicated. Deep sequencing metrics are depicted, including length (in base pairs) of the SARS-CoV-2 isolates sequenced. (B) A Maximum Likelihood phylogenetic tree was constructed using the whole genome SARS-CoV-2 consensus sequences obtained from all five SARS-CoV-2 isolates (in red) rooted with the Wuhan-Hu-1 NC_045512 sequence and 21 whole genome SARS-CoV-2 sequences, representative of viruses considered variants of concern (VOC) by the World Health Organization (downloaded on 30 June 2022 from the GISAID database https://www.gisaid.org). Gray boxes depict clusters associated with SARS-CoV-2 Mu, ancestral (Wuhan), Delta, and Omicron BA.1 variants. Bootstrap resampling (1,000 data sets) of the multiple alignments tested the statistical robustness of the trees, with percentage values above 75% indicated by an asterisk. s/nt, substitutions per nucleotide. (C) Classification of the five SARS-CoV-2 whole genome sequences using [a] Nextstrain (https://nextstrain.org/ncov/, accessed on 30 April 2020), [b] PANGO Lineages (https://cov-lineages.org/index.html, accessed on 30 April 2020), and [c] GISAID database (https://www.gisaid.org/, accessed on 30 April 2020.

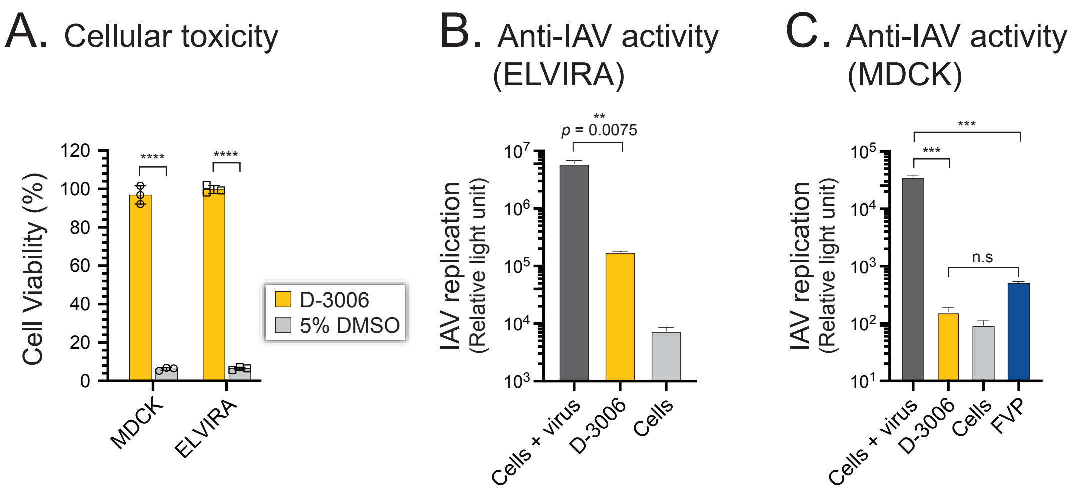

Supplementary Figure 6. Cellular toxicity and anti-IAV acitivyt of D-3006.
(A) The viability of MDCK and ELVIRA Flu A cells exposed to 50 µg/ml of D-3006 or 5% DMSO (control) was determined in triplicate. Susceptibility of Influenza A virus A/Mallard/Alberta/287/2012 (H1N1) strain to 50 µg/ml of D-3006 or 10 µM favipiravir (FVP) using (B) the ELVIRA Flu A cells, which express the firefly luciferase gene in response to infection with IAV or (C) virus-yield assay infecting MDCK cells in the presence and abscence of the different agents, then using the cell supernatant to infect ELVIRA Flu A cells as reporter cells. Depicted values represent medians +/- standard deviations (SD). Wilcoxon-Mann-Whitney test was used to compare IAV replication in the presence of D-3006 or FVP to Cells + virus (positive control). n.s., not significant. *** $p < 0.001$.

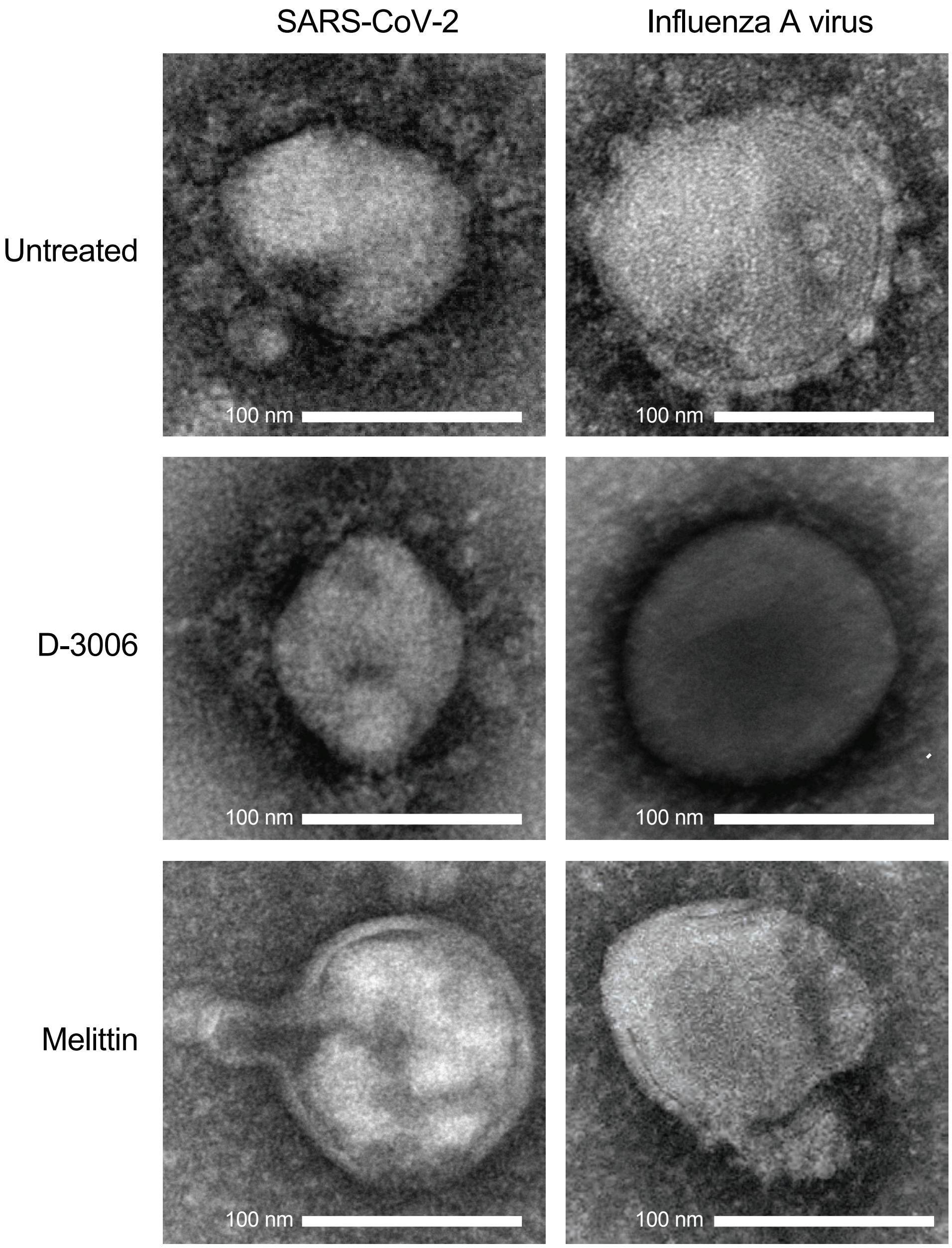

Supplementary Figure 7. Negative-stain transmission electron microscopy analysis.
Aliquots of SARS-CoV-2 hCoV-19/New Zealand/NZ1 or Influenza A virus A/Mallard/Alberta/287/2012 (H1N1) strains were incubated with 100 µg/ml of D-3006, melittin, or 1X DMEM (Untreated) for one hour at 37ºC and 5% CO_2, fixed with 2% formaldehyde, purified (20% sucrose cushion) and prepared for negative staining transmission electron microscopy analysis. Grids were investigated using an Philips CM100 transmission electron microscope at the Otago Micro and Nanoscale Imaging (OMNI, University of Otago, New Zealand) with an accelerating voltage of 100 KeV.
